# The thiosemicarbazone Me_2_NNMe_2_ induces paraptosis by disrupting the ER thiol redox homeostasis based on protein disulfide isomerase inhibition

**DOI:** 10.1038/s41419-018-1102-z

**Published:** 2018-10-15

**Authors:** Sonja Hager, Katharina Korbula, Björn Bielec, Michael Grusch, Christine Pirker, Markus Schosserer, Lisa Liendl, Magdalena Lang, Johannes Grillari, Karin Nowikovsky, Veronika F. S. Pape, Thomas Mohr, Gergely Szakács, Bernhard K. Keppler, Walter Berger, Christian R. Kowol, Petra Heffeter

**Affiliations:** 1 Institute of Cancer Research and Comprehensive Cancer Center, Department of Medicine I, Medical University of Vienna, Borschkegasse 8a, A-1090 Vienna, Austria; 2 Research Cluster “Translational Cancer Therapy Research”, Vienna, Austria; 3 Institute of Inorganic Chemistry, Faculty of Chemistry, University of Vienna, Waehringer Str. 42, A-1090 Vienna, Austria; 4 Department of Biotechnology, BOKU-University of Natural Resources and Life Sciences, Vienna, Muthgasse 18, A-1190 Vienna Austria; 5 Christian Doppler Laboratory on Biotechnology of Skin Aging, Muthgasse 18, A-1190 Vienna, Austria; 6 Evercyte GmbH, Muthgasse 18, A-1190 Vienna, Austria; 7 Department of Internal Medicine I and Comprehensive Cancer Center, Medical University of Vienna, Lazarettgasse 14, A-1090 Vienna, Austria; 8 Department of Physiology, Faculty of Medicine, Semmelweis University, Tűzoltó utca 37-47, H-1094 Budapest, Hungary; 9 Institute of Enzymology, Research Centre for Natural Sciences, Hungarian Academy of Sciences, Magyar Tudósok körútja 2, H-1117 Budapest, Hungary; 10 Science Consult DI Thomas Mohr KG, Enzianweg 10a, A-2353 Guntramsdorf, Austria

## Abstract

Due to their high biological activity, thiosemicarbazones have been developed for treatment of diverse diseases, including cancer, resulting in multiple clinical trials especially of the lead compound Triapine. During the last years, a novel subclass of anticancer thiosemicarbazones has attracted substantial interest based on their enhanced cytotoxic activity. Increasing evidence suggests that the double-dimethylated Triapine derivative Me_2_NNMe_2_ differs from Triapine not only in its efficacy but also in its mode of action. Here we show that Me_2_NNMe_2_- (but not Triapine)-treated cancer cells exhibit all hallmarks of paraptotic cell death including, besides the appearance of endoplasmic reticulum (ER)-derived vesicles, also mitochondrial swelling and caspase-independent cell death via the MAPK signaling pathway. Subsequently, we uncover that the copper complex of Me_2_NNMe_2_ (a supposed intracellular metabolite) inhibits the ER-resident protein disulfide isomerase, resulting in a specific form of ER stress based on disruption of the Ca^2+^ and ER thiol redox homeostasis. Our findings indicate that compounds like Me_2_NNMe_2_ are of interest especially for the treatment of apoptosis-resistant cancer and provide new insights into mechanisms underlying drug-induced paraptosis.

## Introduction

**Fig. 1:**
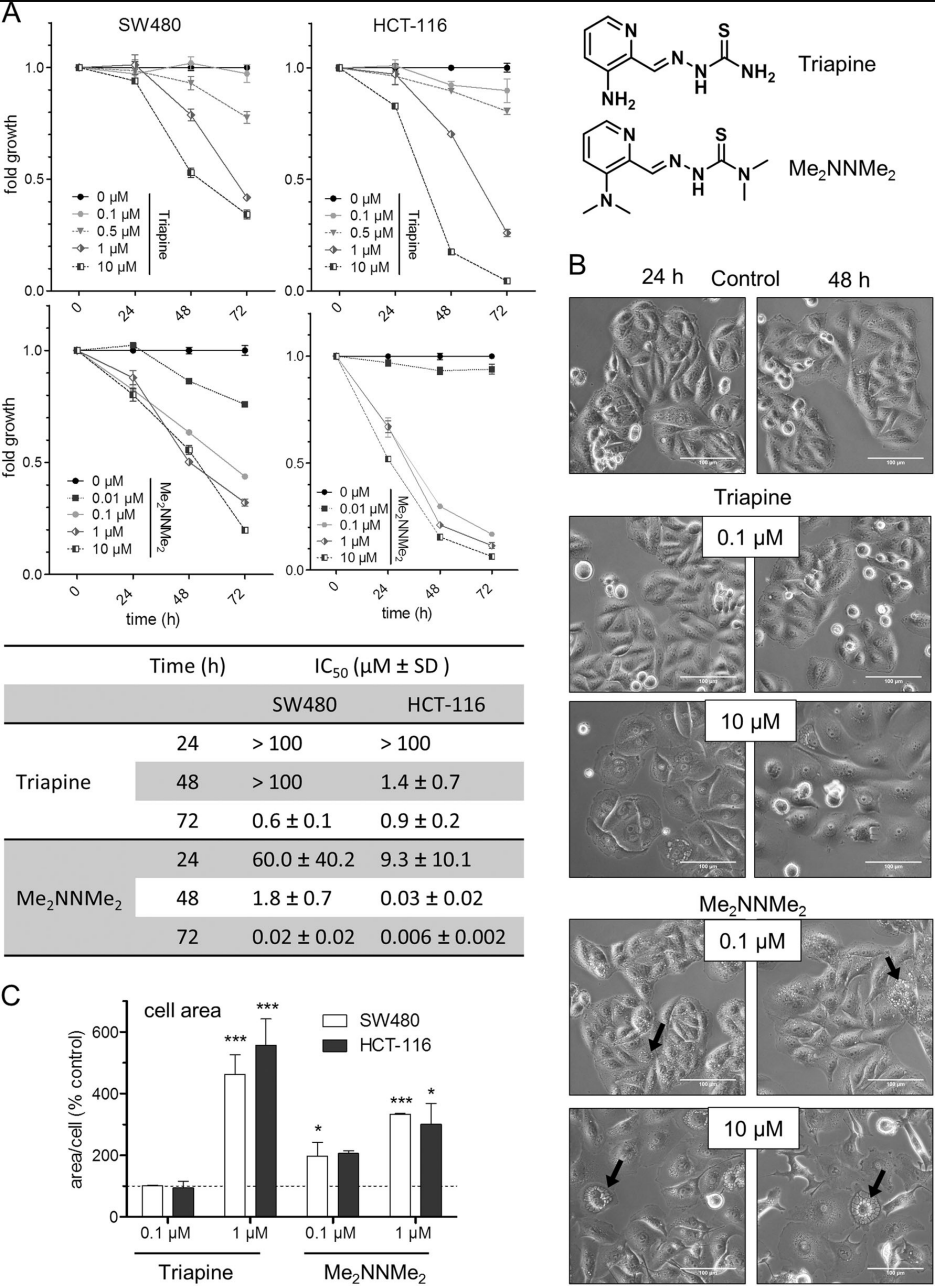
Activity of Triapine and its derivative Me_2_NNMe_2_. **a** Time-dependent cell viability of SW480 and HCT-116 cells treated with either Triapine or Me_2_NNMe_2_, determined by MTT assay after 24, 48, and 72 h. Values given in the graph are the mean ± standard deviation of triplicates from one representative experiment out of three, normalized to the untreated control of the same time-point. IC_50_ values (µM) ± standard deviations (SD) are given in the table . **b** Morphological changes in SW480 cells induced by 24 and 48 h treatment with the indicated concentrations of Triapine or Me_2_NNMe_2_. Cytoplasmic vacuoles were mainly seen with Me_2_NNMe_2_ (arrows). Scale bar: 100 µm. **c** Increase in cell size of SW480 and HCT-116 cells treated with the indicated concentrations of Triapine and Me_2_NNMe_2_ for 48 h

α-*N*-Heterocyclic thiosemicarbazones (TSCs) are a promising class of therapeutics, which have been extensively investigated for their anticancer activity^1,2^. The most prominent and best-studied drug candidate is 3-aminopyridine-2-carboxaldehyde TSC, also known as Triapine. Triapine displayed promising results in clinical phase I and II trials against hematological cancers^3–6^ and has also been tested against diverse solid tumors^7,8^. In addition, several new TSC derivatives have been developed over the last years. Two of them, namely Coti-2 and DpC, have recently entered clinical phase I trials (www.clinicaltrials.gov). Coti-2, DpC as well as the predecessor Dp44mT showed highly improved anticancer activities compared to Triapine with IC_50_ values in the nanomolar concentration range (hence, called "nanomolar TSCs")^9,10^. Our group has recently synthesized a new nanomolar TSC derivative, Me_2_NNMe_2_, characterized by dimethylation of both primary amino groups of the Triapine molecule(Fig. 1)^2,11^.

Based on promising clinical trials, it is of interest to better elucidate the reasons for the greatly improved anticancer activity of nanomolar TSCs. There are several indications that nanomolar TSCs differ in their mode of action from Triapine^2,12,13^. In particular, their interaction with intracellular copper ions might be important, as intracellularly formed copper complexes have been suggested to be the active metabolites of nanomolar TSCs^12–14^. In this regard, during our recent studies, we have discovered that treatment with Me_2_NNMe_2_ as well as Dp44mT resulted in the formation of perinuclear cytoplasmic vesicles^11^ that are characteristic for paraptosis, a recently described new type of programmed cell death^15,16^. Further hallmarks of paraptosis include mitochondrial swelling and damage, caspase-independent cell death and the absence of membrane blebbing/DNA condensation or fragmentation. Moreover, disruption of endoplasmic reticulum (ER) homeostasis, activation of MAPK signaling as well as protection by the thiol-containing radical scavenger *N*-acetylcysteine (NAC) and the MEK inhibitor U0126 have been reported^15,16^. However, the exact molecular mechanisms underlying paraptosis induction are widely unexplored.

So far, mainly diverse natural compounds have been identified as paraptosis inducers. Interestingly, the list also includes some copper complexes^17–19^, supporting the idea that nanomolar TSCs could also induce this novel form of cell death. Therefore, in this study, we investigated the role of apoptotic and paraptotic cell death in the mode of action of Triapine and Me_2_NNMe_2_. Our experiments revealed that treatment with Me_2_NNMe_2_ induces all of the main hallmarks of paraptotic cell death. In addition, we identified the inhibition of the ER-resident protein disulfide isomerase (PDI) as a potential target of the intracellularly formed Me_2_NNMe_2_ copper metabolite.

## Results

### Anticancer activity of Triapine and Me_2_NNMe_2_

Cytotoxicity and morphological changes induced by Triapine and Me_2_NNMe_2_ were investigated in SW480 and HCT-116 cells at different time points (Fig. 1a). In general, HCT-116 cells proved to be more sensitive to TSC treatment than SW480. Moreover, in accordance with previous results^11^, double-dimethylation of Triapine resulted in markedly higher activity in a time-dependent manner. The two drugs had distinct effects on cell morphology, as shown in Fig. 1b, c. Especially, Triapine-treated cells were characterized by increased cell area (up to 500%) and flattening (Fig. 1c). In contrast, treatment with Me_2_NNMe_2_ led to formation of cytoplasmic vesicles (see black arrows in Fig. 1b), which dose- and time-dependently increased in size and number (Fig. 1b, Suppl. Figure 1). These observations were consistent in both cell lines. Comparable vesicle formation was also observed with the other nanomolar TSCs, DpC, Dp44mT, and Coti-2 (Suppl. Figure 2).

### Me_2_NNMe_2_ accumulation in the ER-derived vesicles

**Fig. 2:**
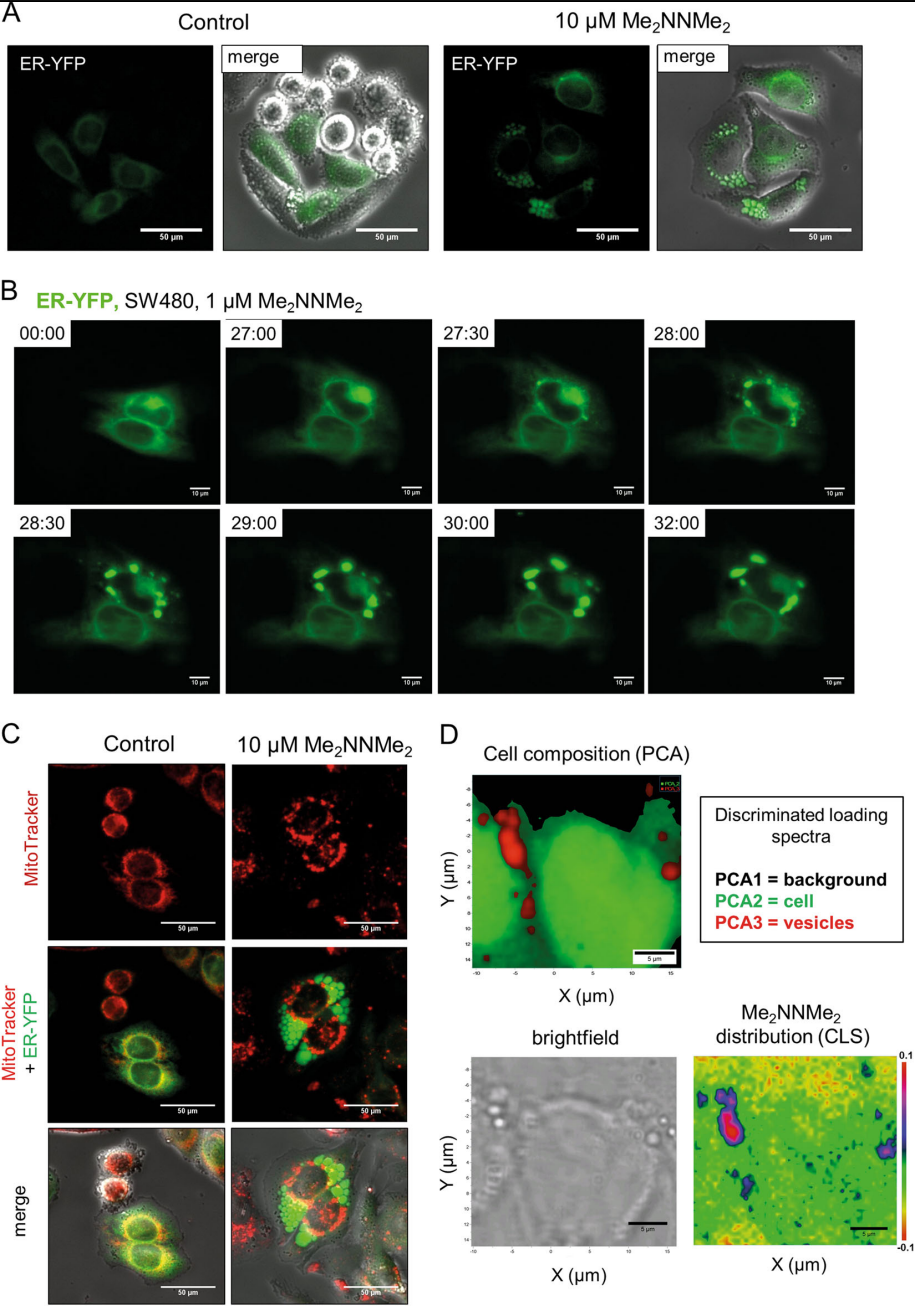
Me_2_NNMe_2_ accumulation in the ER-derived vesicles. **a** Representative fluorescence microscopy images and overlaid differential interference contrast images of the ER lumen of ER-YFP-transfected SW480 cells treated with 10 µM Me_2_NNMe_2_ for 24 h (scale bar: 50 µm). **b** Life-cell fluorescence imaging of ER-located YFP-transfected SW480 cells treated with 1 µM Me_2_NNMe_2_. Time after treatment is indicated as hh:mm (scale bar: 10 µm). **c** Representative fluorescence microscopy images of mitochondria (MitoTracker) showing no overlap with vesicles in ER-YFP-transfected SW480 cells treated with 10 µM Me_2_NNMe_2_. (scale bar: 50 µm). **d** Raman microspectroscopy of SW480 cells treated with 10 µM Me_2_NNMe_2_ for 24 h. Principal component analysis (PCA) of Raman spectra can differentiate between background (black), cell (green) and vesicles (red). CLS fitting of Me_2_NNMe_2_ Raman spectrum to the spectral map of the cell revealed accumulation of the drug inside the vesicles

Several groups have reported that paraptosis induction is associated with the appearance of cytoplasmic vesicles originating from the ER^15,16^. To investigate whether the cytoplasmic vesicles seen in Me_2_NNMe_2_-treated cells also arise from the ER, transfection experiments with ER-localized YFP were performed (Fig. 2a). As visualized by live-cell microscopy, ER-derived vesicles formed around the nucleus and rapidly increased in size (by fusion) (Fig. 2b). Moreover, no overlap of these vesicles with mitochondria or lysosomes was found (Fig. 2c and Suppl. Figure 3). Consequently, we concluded that the observed cytoplasmic vesicles after Me_2_NNMe_2_ treatment originated solely from the ER.

Mapping cells by Raman microspectroscopy and subsequent principal component analysis (PCA) revealed a unique biochemical composition of these vesicles compared to the rest of the cell (Fig. 2d). Component spectra suggested enrichment of lipids (bands at ~1295 cm^−1^, 1435–1480 cm^−1^, and ~1650 cm^−1^) in these vesicles, while bands corresponding to nucleic acids (~715 cm^−1^, ~775 cm^−1^, ~1090 cm^−1^, and ~1570 cm^−1^) were weaker compared to the rest of the cell (Suppl. Figure 4A)^20^. Furthermore, classical least squares (CLS) fitting of the spectrum of the pure substance (Suppl. Figure 4B) to the Raman map revealed that Me_2_NNMe_2_ appears to accumulate in these vesicles (Fig. 2d), indicating that the compound might have its intracellular target in the ER.

### Impact of the TSCs on mitochondrial integrity

**Fig. 3:**
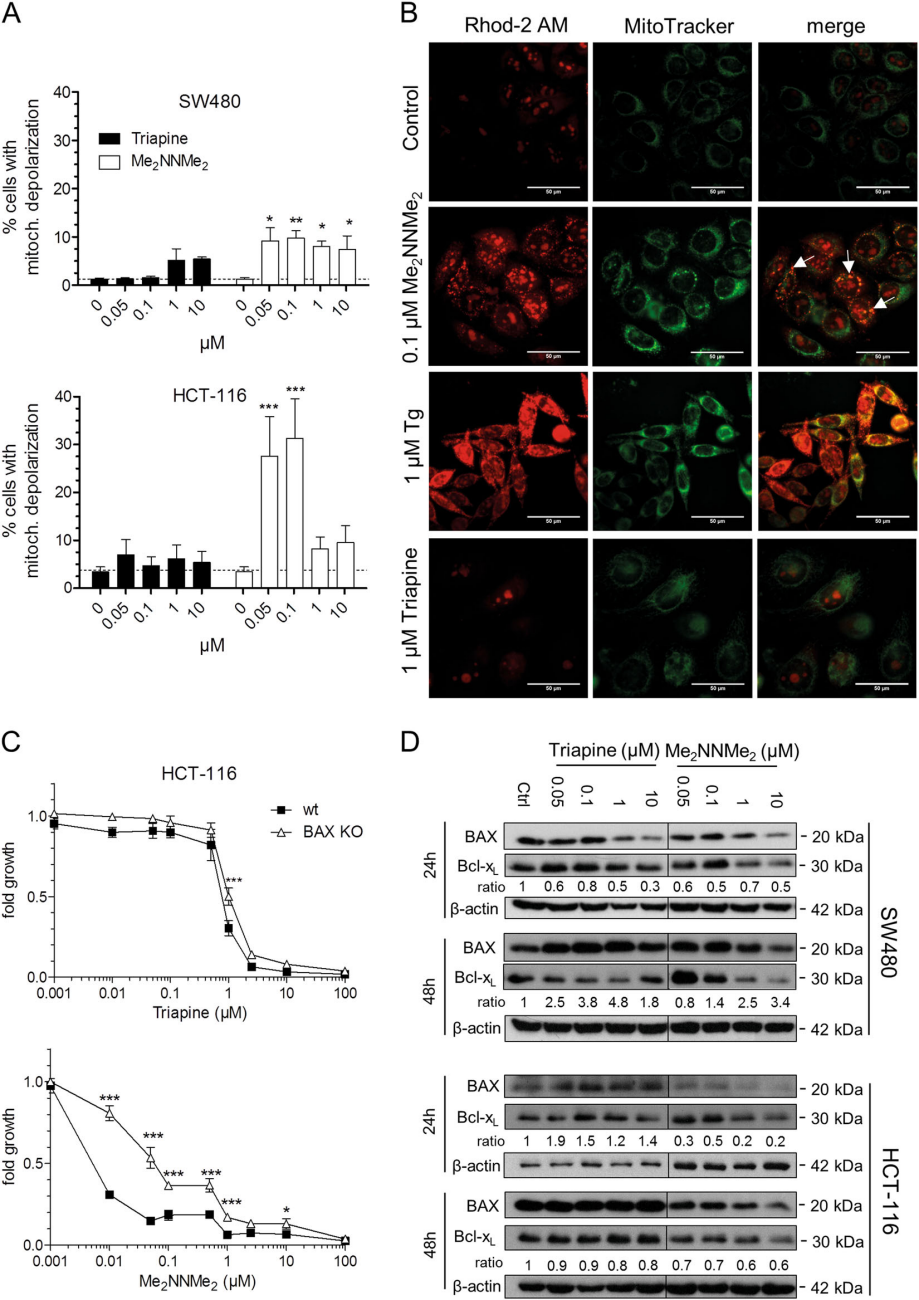
Mitochondrial involvement in the activity of Triapine and Me_2_NNMe_2_. **a** Mitochondrial membrane potential depolarization measured by the percentage of cells with decreased JC-1 fluorescence (red). SW480 or HCT-116 cells were treated with the indicated concentrations of Triapine or Me_2_NNMe_2_ for 24 h. Values given are the mean ± standard deviation of three independent experiments. **b** Fluorescence microscopy of increased calcium levels (Rhod-2 AM in red) specifically in the mitochondria (MitoTracker in green) after thapsigargin (Tg, 1 µM), Me_2_NNMe_2_ (0.1 µM) or Triapine (1 µM) treatment of SW480 cells for 48 h (scale bar: 50 µm). White arrows indicate co-localization. **c** Cell viability of HCT-116 wild-type (wt) and BAX knockout (KO) cells measured by MTT after 72 h treatment with indicated concentrations of Triapine or Me_2_NNMe_2_. Values given are the mean ± standard deviation of triplicates of one representative experiment out of three. **d** Western blot analysis of BAX and Bcl-x_L_ expressed by SW480 and HCT-116 cells treated with Triapine or Me_2_NNMe_2_ for 24 or 48 h. The ratio of BAX to Bcl-x_L_ is given below the respective bands. β-actin was used as a loading control. Significance was calculated to control with one-way (**a**) and to wt cells with two-way (**c**) ANOVA and Bonferroni’s multiple comparison test (***p < 0.001, **p ≤ 0.01, *p ≤ 0.05)

Paraptotic cell death is frequently associated with changes of mitochondrial morphology and functionality^21–27^. Consequently, JC-1 staining was conducted to evaluate the impact of both drugs on mitochondrial membrane potential. Upon treatment with Triapine, only slight, non-significant effects were detected in both cell lines (Fig. 3a), while Me_2_NNMe_2_ had a profound impact. In detail, in SW480 cells, at all investigated concentrations ~10% of the cells displayed depolarized mitochondria. In contrast, 30% of HCT-116 cells showed mitochondrial depolarization at 0.05 and 0.1 µM Me_2_NNMe_2_, which decreased to about 10% at higher concentrations. In parallel to mitochondrial depolarization, Me_2_NNMe_2_, but not Triapine, induced mitochondrial fragmentation or swelling (a main hallmark of paraptosis) already at 0.1 µM (Suppl. Figure 5). In order to investigate whether this observed swelling is accompanied by increased intra-mitochondrial Ca^2+^ levels, Rhod-2 AM stains were performed. Indeed, distinct accumulation of mitochondrial Ca^2+^ together with organelle swelling was observed in Me_2_NNMe_2_-exposed cells (Fig. 3b). In contrast, thapsigargin, a well-known SERCA (ER-localized Ca^2+^ ATPase) inhibitor and ER stress inducer, initiated mitochondrial Ca^2+^ accumulation but no organelle swelling. Together with the lack of organelle swelling, Triapine had also no impact on mitochondrial Ca^2+^ levels (Fig. 3b).

In agreement with the suggested contribution of mitochondria to Me_2_NNMe_2_ activity, HCT-116 cells with a BAX knockout^18^ were (in contrast to Triapine) significantly less sensitive to the methylated derivative (Fig. 3c). Interestingly, Me_2_NNMe_2_ activity was accompanied by a decrease of both pro-apoptotic BAX as well as anti-apoptotic Bcl-x_L_ protein levels in BAX wild-type cells, which argues against induction of apoptosis via the intrinsic (mitochondrial) pathway (Fig. 3d). Taken together, this indicates that Me_2_NNMe_2_ distinctly impacts on mitochondrial integrity already at very low drug concentrations and disruption of mitochondrial Ca^2+^ homeostasis is a key event in Me_2_NNMe_2_-induced paraptosis.

### Caspase independence of Me_2_NNMe_2_ anticancer activity

**Fig. 4:**
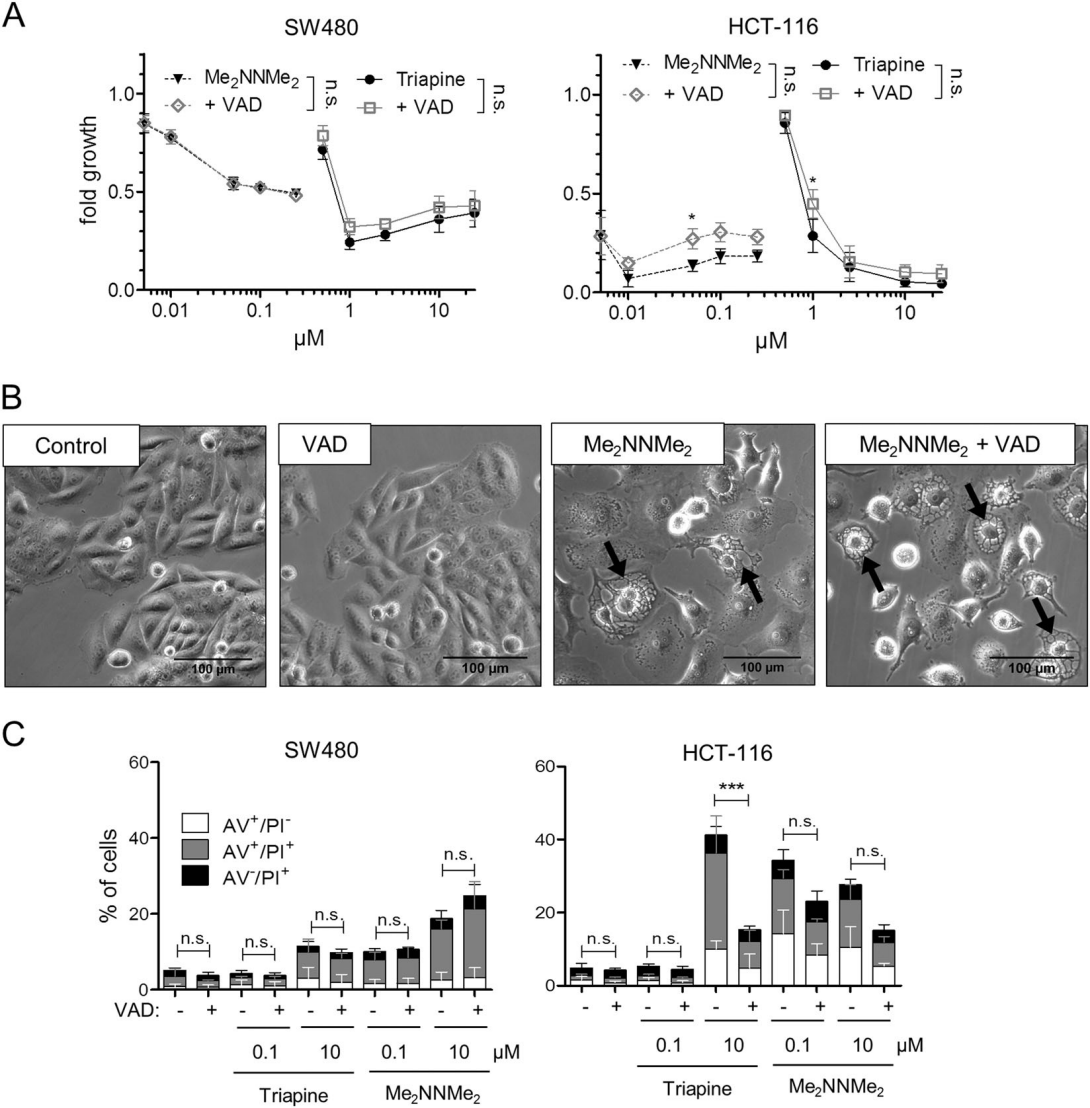
Induction of different cell death characteristics by Triapine and Me_2_NNMe_2_. **a** Cell viability measured by MTT assay of SW480 and HCT-116 cells after 72 h treatment with the indicated concentrations of Triapine (full lines) or Me_2_NNMe_2_ (dotted lines) alone or in combination with 10 µM z-VAD-FMK (VAD, gray lines). Values given are the mean ± standard deviation of triplicates from one representative experiment out of three. **b** Phase-contrast microscopy images of SW480 cells treated with 10 µM Me_2_NNMe_2_ or 25 µM z-VAD-FMK as well as the combination (scale bar: 100 µm). **c** Percentage of annexin V-positive (AV^+^) and/or PI^+^ SW480 or HCT-116 cells detected by flow cytometry after 48 h of Triapine or Me_2_NNMe_2_ treatment in combination with 10 µM z-VAD-FMK (VAD). Values given are the mean ± standard deviation of three independent experiments. For calculation of significance AV^+^ cell fractions (AV^+^/PI^−^, AV^+^/PI^+^) were added. Significance to control was calculated by two-way (**a**) or one-way (**c**) ANOVA and Bonferroni’s multiple comparison test using GraphPad Prism software (***p < 0.001, **p ≤ 0.01, *p ≤ 0.05)

As paraptosis is often described as a caspase-independent process^15,16^, as a next step the impact of the pan-caspase inhibitor z-VAD-FMK on the activity of the two TSCs was investigated. As shown in Fig. 4a, there was no relevant effect of z-VAD-FMK on the anticancer activity of the tested TSCs, in contrast to TRAIL, which was used as a positive control (Suppl. Figure 6). In addition, treatment with the pan-caspase inhibitor did not prevent the formation of cytoplasmic vesicles induced by Me_2_NNMe_2_ (Fig. 4b). To confirm the caspase independence of Me_2_NNMe_2_-induced cell death, annexin V (AV) stains were performed in the presence and absence of the pan-caspase inhibitor (Fig. 4c). Caspase inhibition had no significant impact (calculated to control by one-way ANOVA and Bonferroni’s multiple comparison test) on the AV^+^ cell fractions after Me_2_NNMe_2_ treatment in both cell lines. In contrast, Triapine-induced cell death in HCT-116 was strongly diminished upon addition of z-VAD-FMK, suggesting cell line-dependent apoptosis induction by this compound.

### The role of MAPKs in Me_2_NNMe_2_-induced paraptosis

**Fig. 5:**
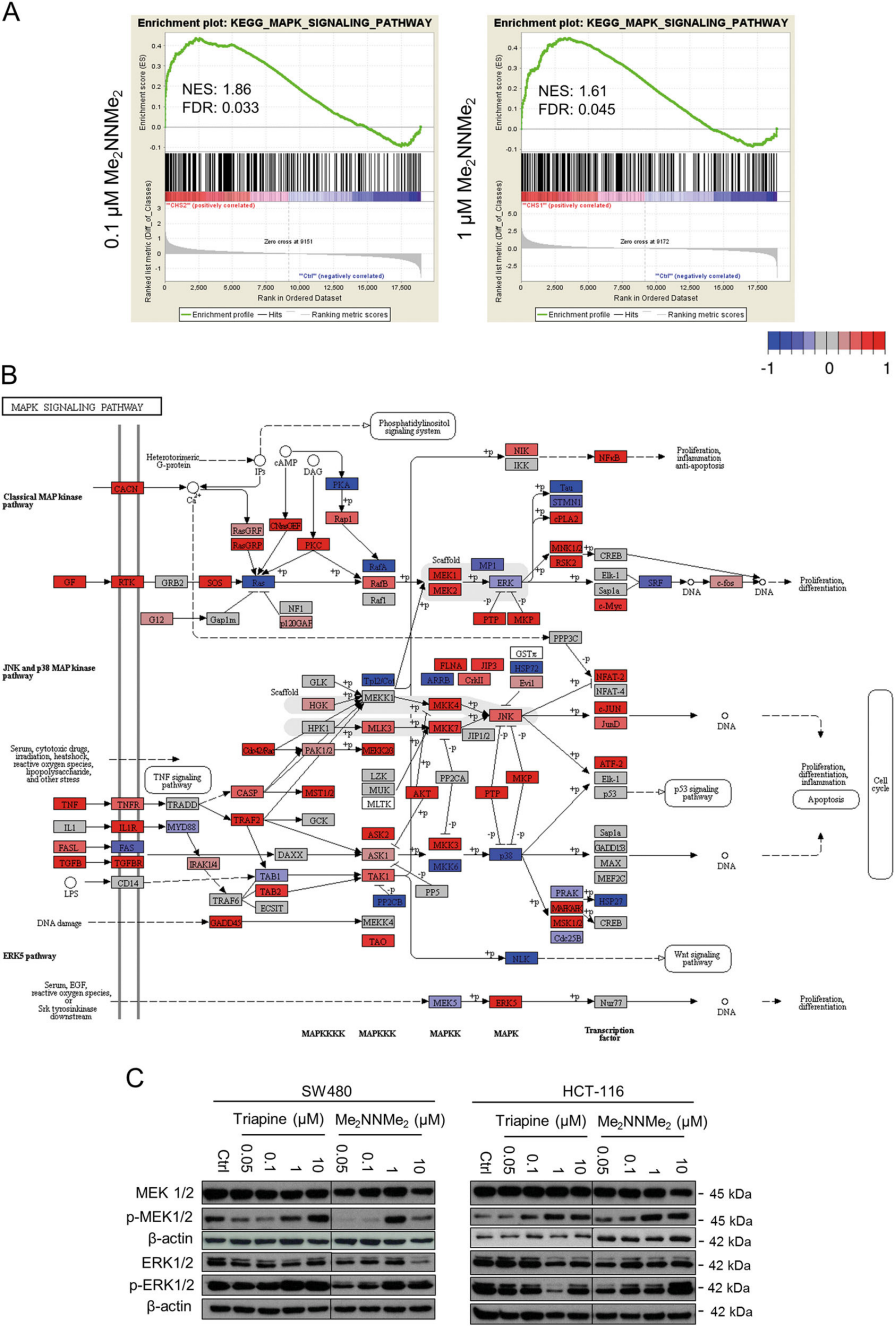
Role of the MAPK pathway in the activity of Triapine and Me_2_NNMe_2_. **a** GSEA from whole-genome gene expression data revealed significant enrichment of genes in the “MAPK signaling pathway” gene set in SW480 cells treated with 0.1 or 1 µM Me_2_NNMe_2_ compared to untreated cells. Normalized enrichment score (NES) and false discovery rate (FDR) are given. **b** Illustration of genes up- (red) or down-regulated (blue) in the KEGG-derived “MAPK signaling pathway” of Me_2_NNMe_2_ (1 µM)-treated compared to untreated SW480 cells using whole-genome gene expression data. **c** Western blot analysis of MEK1/2 and ERK1/2 as well as their phosphorylated protein levels in SW480 and HCT-116 cells treated with indicated concentrations of Triapine and Me_2_NNMe_2_ for 24 h. β-actin was used as a loading control

There are indications that MAPK signaling plays an important role in the execution of paraptotic cell death^16,28^. However, whether and how Me_2_NNMe_2_ activity impacts on this pathway is so far unknown. Consequently, as a first step, we compared gene signatures of whole-genome gene expression arrays performed with 0.1 µM and 1 µM Me_2_NNMe_2_ treatment or untreated cells. Gene set enrichment analysis (GSEA) of these data showed significant upregulation of MAPK signaling pathway genes in treated as compared to untreated cells at both concentrations (Fig. 5a). A more detailed illustration of the genes up- (red) or down- (blue) regulated in this KEGG pathway is shown in Fig. 5b. When comparing these mRNA data with Western blot analysis of MEK and ERK, interestingly, both Triapine and Me_2_NNMe_2_ treatment had a tendency to stimulate the MAPK signaling at higher drug concentrations (Fig. 5c). However, at lower doses strongly reduced phosphorylation (especially of MEK1/2) was observed, indicating that stimulation of the MAPK pathway could be due to a compensatory feedback loop.

**Fig. 6:**
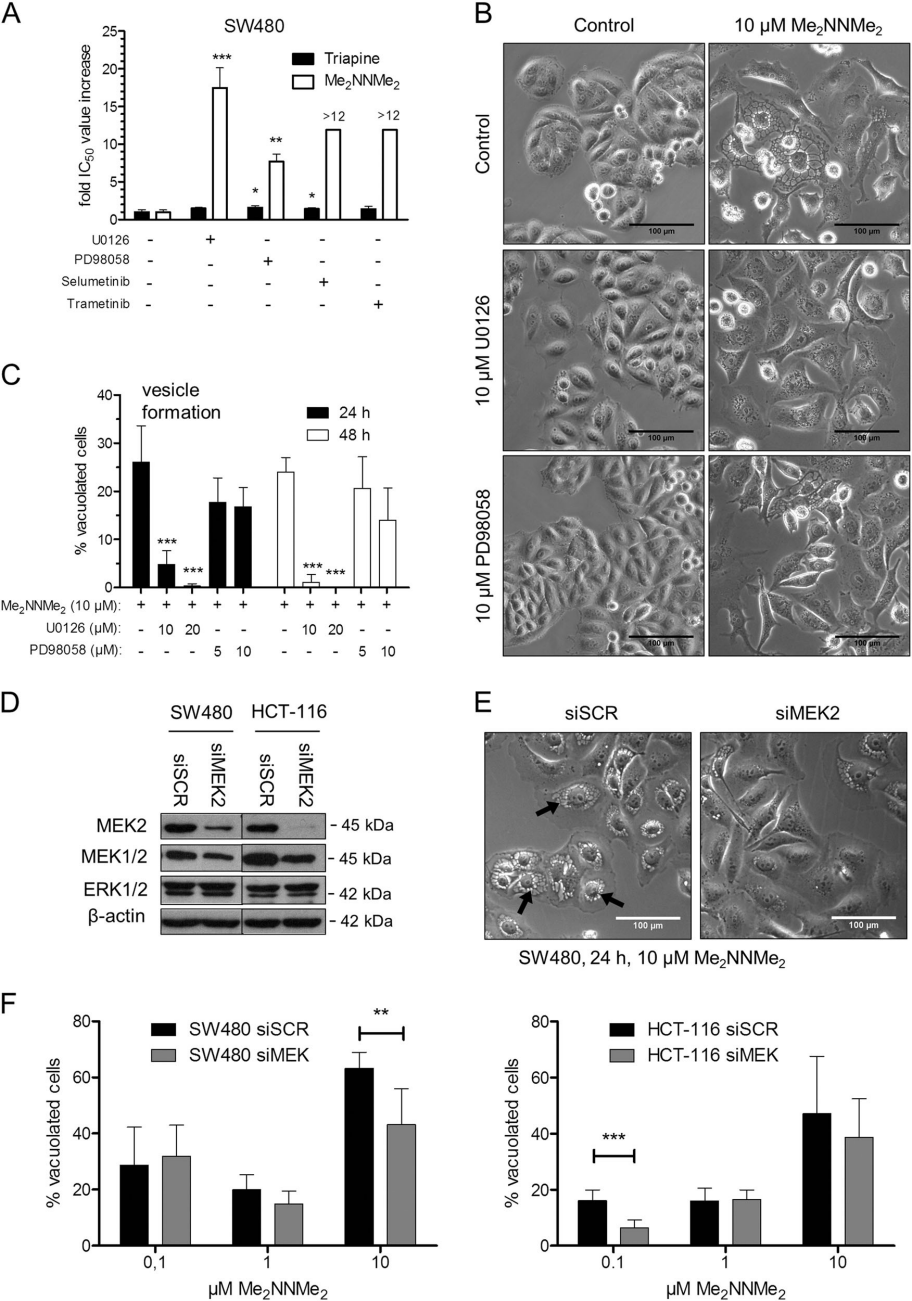
MEK1/2 inhibition affects Me_2_NNMe_2_-induced cell death. **a** Impact of MEK1/2 inhibitors U0126 (5 µM), PD98058 (5 µM), selumetinib (50 nM) or trametinib (100 nM) on viability of Triapine- or Me_2_NNMe_2_-treated SW480. Change in viability is given as mean fold IC_50_ value increase ± standard deviation compared to Triapine or Me_2_NNMe_2_ treatment alone, measured by three independent MTT viability experiments. **b** Representative phase-contrast microscopy images of SW480 cells treated with Me_2_NNMe_2_ (10 µM) and U0126 (20 µM) or PD98058 (10 µM) as well as the combinations for 48 h (scale bar: 100 µm). **c** Percentage of vacuolated cells counted from phase-contrast microscopy images seen in **b**. Values given are the mean ± standard deviation of three images with at least 30 cells in total. Significance to single treatment was calculated by one-way ANOVA and Bonferroni’s multiple comparison test (***p < 0.001, **0.001 ≥ *p* ≤ 0.01, *0.01 ≥ *p* ≤ 0.05). **d** Protein expression detected by Western blot of MEK2, MEK1/2, and ERK1/2 in SW480 and HCT-116 cells after 48 h gene silencing with scrambled (siSCR) or MEK2 (siMEK2) siRNA. β-actin was used as a loading control. **e** Representative images of SW480 cells transfected with siSCR or siMEK2 and treated with 10 µM Me_2_NNMe_2_ for 24 h (scale bar: 100 µm). **f** Percentage of cell vacuolization of SW480 or HCT-116 cells transfected with siSCR or siMEK2 and treated with the indicated concentrations of Me_2_NNMe_2_ for 24 h. Values given are the mean ± standard deviation of several regions of two experiments. Significance to siSCR was calculated by Student's *T*-test (***p < 0.001, **p ≤ 0.01)

To gain more insight into the role of the MAPK pathway in the activity of our TSCs, several MEK inhibitors (U0126, PD98058, trametinib, and selumetinib) with different affinities for MEK1 and MEK2 were used. As seen in Fig. 6a and Suppl. Table 1, all inhibitors were able to protect cells against Me_2_NNMe_2_-induced cytotoxicity. However, only U0126 distinctly reduced vesicle formation in Me_2_NNMe_2_ (Fig. 6b, c). The effects of U0126 were also confirmed in HCT-116 cells (data not shown). In contrast to Me_2_NNMe_2_, Triapine activity was largely unaffected by the MEK inhibitors. As U0126 is the only inhibitor that inhibits MEK1 and 2 to a similar extent (while the others have a stronger preference for MEK1), we hypothesized that MEK2 could have a special role in Me_2_NNMe_2_ activity. To further evaluate this hypothesis, knockdown experiments using siRNA against MEK2 were performed (Fig. 6d). Indeed, further analysis revealed that Me_2_NNMe_2_-induced vacuolization decreased upon MEK2 knockdown (Fig. 6e, f) confirming the importance of this protein in the formation of paraptotic vesicles by Me_2_NNMe_2_. Noteworthy, also induction of vesicles and anticancer activity of other nanomolar TSC (DpC, Dp44mT, and Coti-2) could be inhibited by U0126 (Suppl. Figure 7), indicating induction of paraptotic cell death also with these TSCs.

### Me_2_NNMe_2_-induced ER stress based on disturbed ER thiol redox homeostasis

**Fig. 7:**
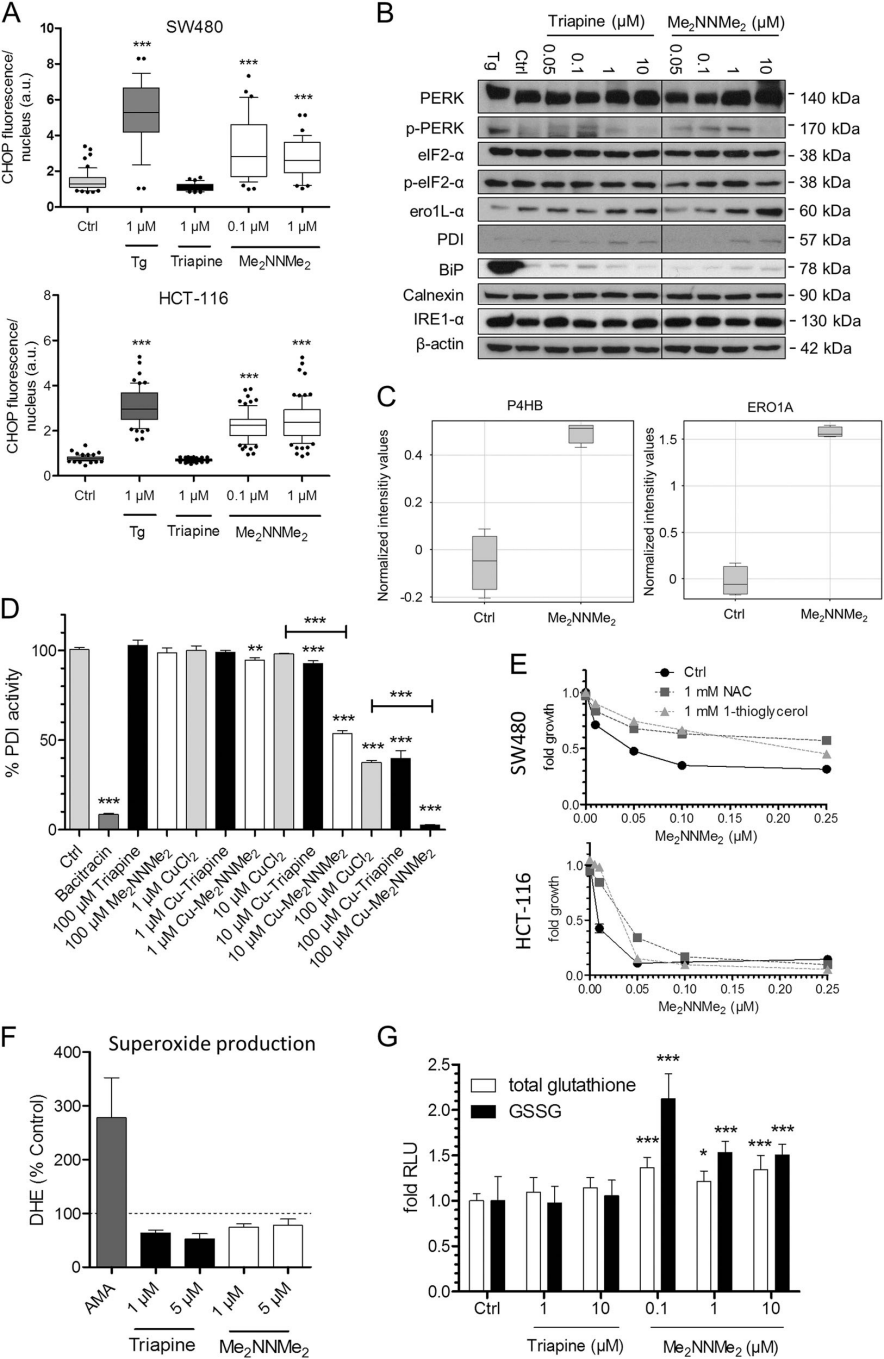
ER stress and disruption of thiol redox homeostasis by Me_2_NNMe_2_ treatment. **a** Quantification of immunofluorescence intensities in the nucleus of the ER stress marker CHOP in SW480 and HCT-116 cells treated with 1 µM thapsigargin (Tg), 1 µM Triapine or 0.1 and 1 µM Me_2_NNMe_2_ for 24 h. Values given are the mean intensities ± the interquartile range and 10 and 90 percentile whiskers of one representative experiment out of three. **b** Western blot analysis of various ER stress proteins expressed by SW480 cells treated with indicated concentrations of Triapine and Me_2_NNMe_2_ for 24 h. β-actin was used as a loading control and Tg (1 µM) as positive control for ER stress. **c** mRNA expression levels for PDI (P4HB) and ero1L-α (ERO1A) in treated (1 µM Me_2_NNMe_2_) or untreated SW480 cells were assessed by whole-genome gene expression microarrays. Two independent P4HB oligonucleotides were spotted on the array and gave comparable results. Data for oligonucleotide A_23_P107412 is shown. Normalized values of four replicates indicate upregulation of PDI and ero1L-α mRNA in treated compared to untreated cells. **d** PDI reduction activity in the presence of Triapine, Me_2_NNMe_2_ as well as their copper complexes was measured by PROTEOSTAT PDI assay kit. Bacitracin (1 mM) was used as a positive PDI inhibition control. Values given are the mean ± standard deviation of triplicates of one representative experiment out of two. **e** Cell viability measured by MTT assay of SW480 or HCT-116 cells after 72 h treatment of indicated concentrations of Me_2_NNMe_2_ alone as well as in combination with *N*-acetylcysteine (NAC) or 1-thioglycerol. Values given are the mean ± standard deviation of triplicates of one representative experiment out of three. **f** Superoxide production measured by flow cytometry of DHE fluorescence in HL-60 cells treated with indicated concentrations of Triapine and Me_2_NNMe_2_ for 45 min. Antimycin A (AMA) was used as positive control. **g** Detection of total and oxidized glutathione (GSSG) by fold increase to control of luminescence in SW480 cells treated with indicated concentrations of Triapine and Me_2_NNMe_2_ for 24 h. Significance to control (or CuCl_2_) was calculated with one-way ANOVA and Dunnett’s multiple comparison test (***p < 0.001, **p ≤ 0.01, *p ≤ 0.05)

So far, there are only a few hypotheses on the exact mechanisms underlying paraptosis induction. In case of natural products, especially proteasome inhibition resulting in (unfolded) protein stress has been suggested^16,29^. Consequently, paraptosis induction by such drugs is often dependent on active protein synthesis. However, inhibition of protein synthesis (by cycloheximide) had no impact on the activity of Me_2_NNMe_2_ and no difference was observed in the impact on protein ubiquitination levels between Triapine and Me_2_NNMe_2_ (data not shown), suggesting another mode of action. Based on ER localization of Me_2_NNMe_2_ in the Raman microscopy studies together with the profound ER blebbing, we hypothesized that Me_2_NNMe_2_ might have a target in this organelle. In line with this hypothesis, subsequent experiments confirmed a specific form of ER stress especially in Me_2_NNMe_2_-treated cells. In more detail, Me_2_NNMe_2_ (but not Triapine) treatment resulted in enhanced nuclear localization of CHOP, an ER stress-induced transcription factor, (Fig. 7a and Suppl Figure 8) together with increased phosphorylation of its upstream activator PERK (Fig. 7b). In contrast, no changes in other ER stress markers, such as BiP, IRE1α, calnexin, or changes in the phosphorylation of eIF2-α were detected. Remarkably, in contrast to thapsigargin, CHOP-regulated ero1L-α (an ER-specific thiol oxidase) as well as the ER-localized chaperone, isomerase and thiol oxidoreductase PDI were upregulated by both Triapine and Me_2_NNMe_2_ (Fig. 7b). Moreover, our array data showed that the expression of these proteins was also increased on mRNA level upon Me_2_NNMe_2_ treatment (Fig. 7c), indicating increased gene transcription of these CHOP-target genes.

Interestingly, there are reports that PDI is able to bind and reduce copper (although the impact of copper binding on the enzymatic activity is not fully characterized)^30^. As Me_2_NNMe_2_ has strong copper-binding properties and our previous studies already indicated that addition of copper strongly increases the activity of Me_2_NNMe_2_^11^, we hypothesized that our drug or its copper metabolite interferes with the functionality of PDI. Subsequently performed enzyme inhibition assays revealed that, indeed, the copper complex of Me_2_NNMe_2_ (but not of Triapine) had strong PDI-inhibitory potential (Fig. 7d). Noteworthy, the metal-free drugs did not inhibit the enzyme, even at high concentrations, suggesting that prior (intracellular) copper chelation is necessary for PDI inhibition. Similar activity was also detected with the copper complexes of DpC and Dp44mT (Suppl. Figure 9).

PDI plays a key role in the ER thiol redox homeostasis by forming and rearranging disulfide bonds during protein folding. In this process, PDI oxidizes unfolded target proteins with the help of oxidized thiol-containing molecules, such as GSSG or ero1L-α, thereby resulting in the reduction of these molecules^31^. To gain more insight into the role of the ER thiol redox homeostasis in the mode of action of Me_2_NNMe_2_, co-incubation experiments with the thiol-containing antioxidants NAC and 1-thioglycerol were performed. Indeed, both compounds protected the cells from Me_2_NNMe_2_ (but not Triapine)-induced cytotoxicity (Fig. 7e and Suppl. Figure 10). In addition, NAC also reduced anticancer activity induced by DpC, Dp44mT and Coti-2 (Suppl. Figure 7C). Noteworthy, these Me_2_NNMe_2_-induced effects were not based on enhanced global superoxide (Fig. 7f) or ROS^11^ levels but coincided with increased glutathione and especially GSSG levels (Fig. 7g). This suggests that nanomolar TSCs induce either a very local, organelle specific form of ROS or ROS generation does not play a major role in their anticancer activity.

**Fig. 8:**
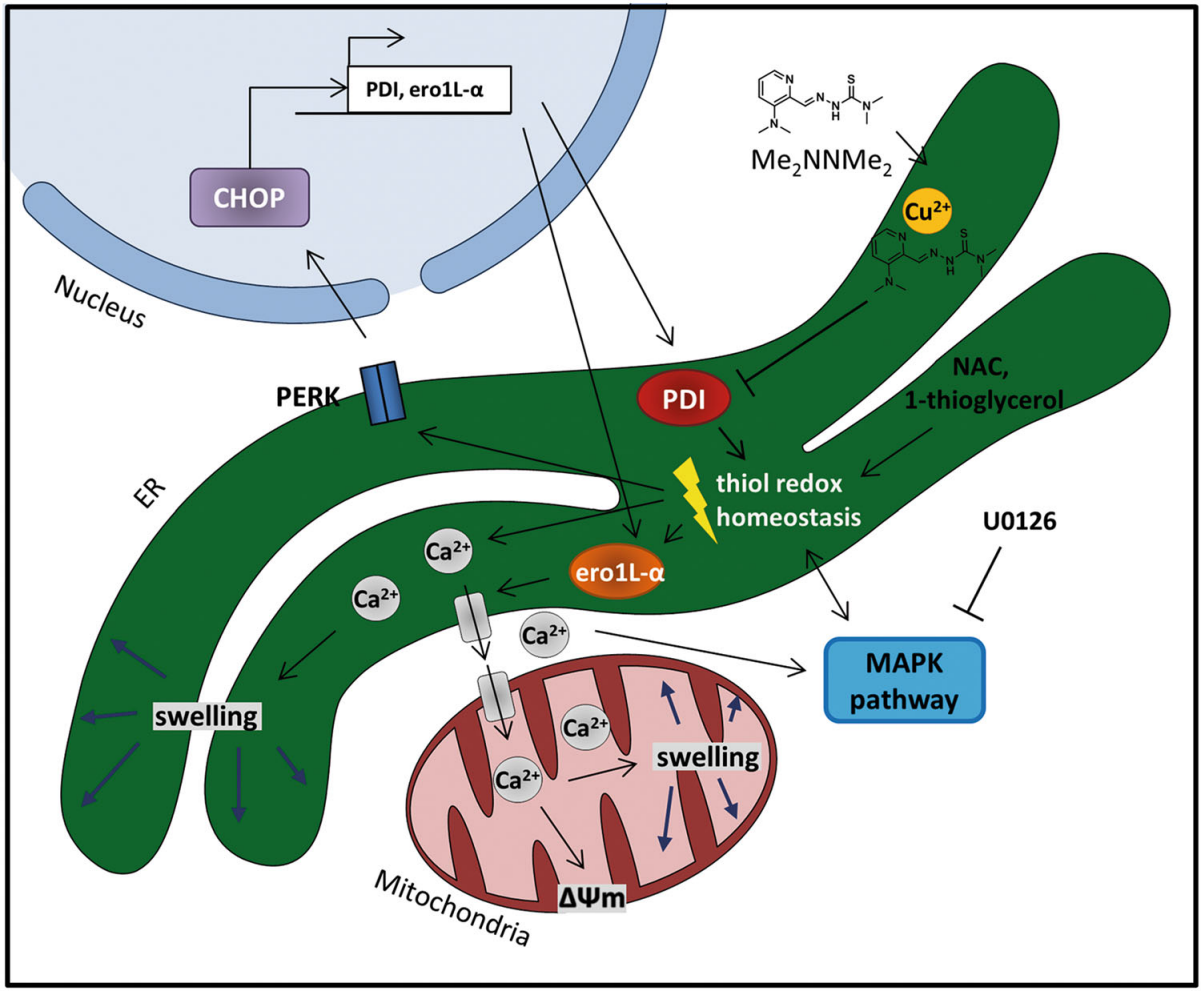
Proposed mechanism of Me_2_NNMe_2_-induced paraptosis. Me_2_NNMe_2_ accumulates in the ER, where it inhibits the reductive potential of PDI. This leads to the disruption of the ER thiol redox homeostasis, which in turn activates PERK signaling and release of Ca^2+^ ions from the ER. While PERK activation is followed by CHOP translocation into the nucleus and increased transcription of PDI and ero1L-α, released Ca^2+^ ions are taken up by mitochondria. Prolonged Ca^2+^ imbalance initiates organelle swelling and mitochondrial membrane depolarization. NAC and 1-thioglycerol can ameliorate thiol redox imbalances. MAPKs further regulate Ca^2+^ and thiol redox homeostasis, which can be inhibited by U0126

Taken together, these results indicate that Me_2_NNMe_2_ might form an intracellular copper metabolite with PDI-inhibitory properties, which then results in disturbed ER thiol redox balance and paraptosis induction. The proposed mode of action is shown in Fig. 8.

## Discussion

In anticancer therapy, resistance of cancer cells to apoptosis is a major obstacle to successful treatment and the cause of many cancer-associated deaths^32^. Targeting cancer cells by the induction of paraptosis, a recently discovered alternative caspase-independent cell death pathway^15,16^, offers the opportunity to overcome apoptosis resistance. However, the mechanisms of paraptosis are still not fully understood (and sometimes even contradictory observations have been published^16,33^), making the in-depth investigation of the underlying signaling pathways of high importance. In general, there are several main hallmarks of paraptosis that are widely accepted. Among these, cytoplasmic (ER-derived) vacuolization, mitochondrial swelling/damage, caspase independence together with absence of membrane blebbing as well as DNA condensation/fragmentation, disruption of ER homeostasis, activation of MAPK signaling, protection by NAC and U0126 as well as protein synthesis dependence are most prominent^15,16^.

So far, mainly natural compounds, such as celastrol, curcumin or cyclosporine A, were found to induce paraptosis^16^. In addition, a few synthetic drugs, including some copper complexes^17,19^, have been studied. Here, for the first time, we report about paraptosis induction by TSCs. Initiated by the discovery that treatment with nanomolar TSCs, such as Me_2_NNMe_2_ and Dp44mT, resulted in formation of prominent cytoplasmic vesicles^11^, our aim in the here presented study was to investigate whether treatment with Me_2_NNMe_2_ results in paraptosis or a paraptosis-like cell death. Therefore, we have investigated different pathways and organelles involved in (apoptotic) cell death and paraptosis. Through this approach, we found that indeed Me_2_NNMe_2_ induced paraptotic cell death fulfilling several main hallmarks such as swelling of ER and mitochondria, caspase independence and MAPK activation (probably via MEK2 signaling).

Interestingly, Raman microscopy experiments revealed an accumulation of Me_2_NNMe_2_ in the ER-derived vesicles, suggesting that this compound might directly interfere with ER-resident proteins. Subsequent investigations revealed that Me_2_NNMe_2_ treatment indeed induced a specific form of ER stress. In detail, enhanced nuclear localization of CHOP and PERK phosphorylation were detected. Beside these typical ER stress markers, we additionally observed an upregulation of ero1L-α and PDI, which are both involved in the ER thiol redox homeostasis^34^. Here, especially PDI attracted our attention, as it has been recently described as a copper-binding and -reducing protein^30^. This is of relevance as Me_2_NNMe_2_ (and other nanomolar TCSs like DpC and Dp44mT) have been well characterized for their metal-chelating properties and especially formation of an intracellular copper metabolite has been suggested to be crucial for their anticancer activity^2,11,12,35,36^. Thus, the PDI-inhibitory potential of Triapine, Me_2_NNMe_2_ as well as their copper complexes was investigated. Indeed, the copper complexes of Me_2_NNMe_2_ as well as those of DpC and Dp44mT were able to potently inhibit the enzyme, while the Triapine copper complex as well as the ligands alone were inactive in this assay. Further evidence connecting TSCs to PDI as a potential target can be seen in the overexpression of the PDI family member CaBP1 in a L1210 cell subline selected for resistance to 4-methyl-5-amino-1-formylisoquinoline TSC (MAIQ)^37^. Although this suggests an important role of this protein class in the mode of action of at least some TSCs, no further studies on this topic have been performed so far. Consequently, the exact evaluation of the mechanisms resulting in the PDI inhibition by some copper TSCs is matter of currently ongoing investigations.

In agreement with the PDI inhibition, subsequent analysis showed that Me_2_NNMe_2_ treatment led to an increase of total glutathione levels, especially of its oxidized form (GSSG) and co-incubation with thiol-containing antioxidants such as NAC or 1-thioglyerol had protective effects. A disrupted thiol redox homeostasis would also explain the enhanced levels of PERK phosphorylation and subsequent CHOP translocation into the nucleus, as seen upon Me_2_NNMe_2_ treatment^38,39^. CHOP in turn is a transcription factor, which can initiate the observed increased expression of (among others) PDI and ero1L-α^40–42^. In general, disruption of the ER thiol redox homeostasis has already been discussed as the cause of ER stress and dilation for other paraptotic inducers^16,28,43^. To the best of our knowledge, this is the first report connecting the induction of paraptosis to the inhibition of ER-resident proteins. Thus, the role of ER enzyme inhibition definitely needs to be addressed in detail in further studies.

With regard to the paraptotic signaling process, the observed thiol-based ER stress is in good agreement with the mitochondrial changes observed after Me_2_NNMe_2_ treatment, as it has already been shown that an altered thiol balance leads to Ca^2+^ release from the ER and its uptake by the mitochondria^44,45^. Thus, mitochondria are proposed to function as a buffer system by absorbing released Ca^2 + ^^46^. However, prolonged occurrence of enhanced mitochondrial Ca^2+^ levels ultimately results in organelle swelling and damage, which explains the excessive depolarization of mitochondria induced by Me_2_NNMe_2_ and many other paraptosis inducers^25,27^. Noteworthy, we found that BAX knockout resulted in reduced sensitivity to Me_2_NNMe_2._ This could be explained by previously observed lowered ER Ca^2+^ stores in BAX-deficient cells, which led to reduced Ca^2+^ uptake by mitochondria after release from the ER^47^. In addition, also a link between PDI and BAX/BAK signaling has already been reported^48^. Nevertheless, why this mitochondrial damage in the course of paraptosis does not activate the intrinsic (mitochondrial) pathway of apoptosis is still a matter of discussion and warrants further investigations.

Taken together, in the here presented study, we identified paraptosis induction via disruption of the ER thiol redox homeostasis as a new mode of action in the activity of the highly active nanomolar TSC Me_2_NNMe_2_ and possibly also for other nanomolar TSCs such as DpC, Dp44mT, and Coti-2. Moreover, we suggest the ER-resident PDI as possible new target for members of this compound class, which could make them interesting candidates for the treatment of cancers with deficiencies in apoptosis induction.

## Materials and methods

### Reagents

Triapine and Me_2_NNMe_2_ were synthesized as previously described^11,49^. U0126 was purchased from Calbiochem, z-VAD-FMK from Enzo Life Sciences (New York, USA), 1-thioglycerol, thapsigargin, antimycin A, NAC, PD98059, trametinib and selumetinib from Selleck Chemicals (TX, USA). All other chemicals were from Sigma-Aldrich.

### Cell culture

The following human cell models were used in this study: the colon carcinoma cell lines SW480 (obtained from the American Tissue Culture Collection) as well as HCT-116 and its respective subline with BAX knockout (obtained from B. Vogelstein, John Hopkins University, Baltimore^18^). SW480 cells were cultured in MEME and HCT-116 cell lines in McCoy’s 5a Medium (from Sigma-Aldrich, MO, USA). The cells were cultivated in medium containing 10% fetal calf serum (FCS, PAA, Linz, Austria).

### Transfection

SW480 cells were plated (3 × 10^5^ cells/well) in 6-well plates and allowed to recover for 24 h. Transfection of pEYFP-ER expression plasmid (#632355, Clontech laboratories, USA) encoding a YFP fused to the ER-targeting sequence of calreticulin at the 5′-end and the ER retention sequence KDEL at the 3′-end or with a control plasmid was performed using Lipofectamine 2000 reagent (Invitrogen, CA, USA) according to the manufacturers’ instructions. Medium was changed after 5 h and selection medium containing 1.2 mg/ml G418 was added 24 h after transfection. Expression of YFP in the ER was investigated 48 h later.

### Cell viability assay

The cells were plated (2 × 10^3^ cells/well) in 96-well plates and allowed to recover for 24 h. Then, cells were treated with Triapine or Me_2_NNMe_2_. In combination treatments, the modulator was always added 1 h in advance. Cell viability was measured by the 3-(4,5-dimethylthiazol-2-yl)-2,5-diphenyltetrazolium bromide (MTT)-based vitality assay (EZ4U; Biomedica, Vienna, Austria) as published^50^. GraphPad Prism software was used to calculate cell viability expressed as IC_50_ values calculated from full dose-response curves.

### Fluorescence staining and microscopy

Cells were seeded into 8-well μ-slides (ibidi GmbH, Germany) with 2 × 10^4^ cells/well and left to recover for 24 h. For organelle tracking, the medium was replaced with serum- and phenol red-free medium with 50 nM MitoTracker Red CMXRos, MitoTracker Green FM or LysoTracker Red (Life technologies, Vienna, Austria). For calcium imaging, cells were incubated with 0.5 µM Rhod-2 AM (Abcam, Cambridge, UK) in serum- and phenol red-free medium for 30 min at 4 °C. After 1 h, cells were washed and imaged with the Nikon Eclipse Ti-e fluorescence microscope with differential interference contrast and RFP or GFP filter settings and a sCMOS pco.edge camera. Life-cell imaging was performed in an environmental chamber pre-heated to 37 °C with 5% CO_2_. For non-fluorescence imaging, phase-contrast pictures were taken with the Nikon Eclipse Ti inverted microscope with a Nikon DS-Fi1c camera. Contrast and brightness were adjusted with ImageJ. Cell area was calculated as mean occupied area per cell from at least two different sections in one well at the end of life-cell imaging (48 h) using ImageJ and then normalized to control.

### CHOP immunofluorescence

Cells (2 × 10^4^/well) were seeded in 8-well chamber slides (ibidi GmbH). After 24 h recovery, cells were treated with indicated drug concentrations and fixed with 4% paraformaldehyde for 15 min at room temperature and (after washing with PBS) blocked and permeabilized with 5% FCS, 0.3% Triton X-100 in PBS for 1 h. The primary antibody CHOP (Cell Signaling Technology) was added 1:3200 in 1% BSA and 0.3% Triton X-100 in PBS overnight at 4 °C. After washing with PBS, the cells were incubated with anti-mouse secondary antibody conjugated to AlexaFluor488 (Thermo Fisher, 1:500 in 1% BSA and 0.3% Triton X-100 in PBS) for 1 h. Cells were again washed and counterstained with 4′,6-diamidine-2′-phenylindole dihydrochloride (DAPI; 1 µg/ml) and wheat germ agglutinin (WGA, 10 µg/ml, Vector Laboratories, CA, USA) in PBS for 10 min. The dyes were removed, and the cells mounted in Vectashield mounting medium (Vector Laboratories, CA, USA) with a coverslip. Images were taken with a Zeiss LSM 700 Olympus (Carl Zeiss AG, Oberkochen, Germany) confocal microscope and CHOP fluorescence intensities per nucleus were measured using ImageJ.

### Annexin V/PI stain and detection of mitochondrial membrane potential

Briefly, 2 × 10^5^ cells/well were seeded in 6-well plates. After 24 h recovery, cells were treated for another 24 h with the indicated drug concentrations. Then, cells were either stained with annexin V-APC (AV) and propidium iodide (PI) or with 10 µg/ml JC-1 as previously described^51,52^.

### Protein expression

After drug treatment, total protein lysates were prepared, separated by SDS-PAGE and transferred onto a polyvinylidene difluoride membrane for Western blotting as described previously^50^. The following antibodies were used: Cell Signaling Technology (MA, USA): BAX (#5023), Bcl-x_L_ (#2764), PERK (#5683), phospho-PERK (Thr980, #3179), Calnexin (#2679), eIF2-α (#5324), phospho-eIF2-α (Ser51, #3398), PDI (#3501), ero1L-α (#3264), BiP (#3177), IRE1α (#3294), MEK1/2 (#9126), phospho-MEK1/2 (Ser217/221, #9154), MEK2 (#9125) ERK1/2 (#4695), phospho-ERK1/2 (Thr202/Tyr204, #4370). Sigma-Aldrich: β-actin (AC-15; #A1978). Primary antibodies were used 1:1000. Secondary, anti-mouse (#7076) and anti-rabbit (#7074) horseradish peroxidase-labeled antibodies from Cell Signaling Technologies were used in working dilutions of 1:10,000.

### Gene knockdown by siRNA

Cells were transfected with Xfect^TM^ RNA Transfection Reagent (Clontech Laboratories, CA, USA) using siRNA against MEK2 (Dharmacon, #M-003573-03-0005) or non-targeting siRNA (Dharmacon, #D-001206-13-05) following the manufacturer’s recommendations. Briefly, 3 × 10^5^ SW480 cells/well or 4 × 10^5^ HCT-116 cells/well were seeded in 6-well plates. After 24 h cells were incubated with the siRNAs and transfection polymer in serum-free medium for 4 h. Then, the medium was exchanged and after another 24 or 48 h cells were collected for experiments. Efficacy and specificity of gene silencing was verified at the protein level by Western blot following 48 h siRNA transfection.

### Total-RNA isolation and whole-genome gene expression array

Total RNA from SW480 cells (either untreated or treated for 15 h) was isolated using RNeasy Mini kit (Quiagen) following the manufacturer’s instruction. Transcriptional profiles of cells were determined performing a 4 × 44 K whole-genome oligonucleotide gene expression array (Agilent) as described previously^53^. Normalization was performed in R using the Bioconductor (version 3.7) package “limma”^54^. Whole-genome gene expression array and gene set enrichment analysis (GSEA) were performed as previously described^51^. Visualization of differentially expressed genes in the KEGG database-derived “MAPK signaling pathway” was conducted using the Bioconductor package “pathview”^55^.

### Raman microspectroscopy

Cells (2 × 10^4^ /well) were seeded into 8-well μ-slides with glass-bottom (ibidi GmbH, Germany) and left to recover for 24 h followed by 24 h drug treatment. Subsequently, samples were fixed with 2% formaldehyde in PBS for 5 min. Cells were mapped in PBS using an XploRA INV Raman microscope (Horiba Jobin Yvon, Bensheim, Germany) equipped with a 532 nm solid state laser at 100 mW, 1800 gr/mm grating and CFI Plan APO ×100 NA 1.4 Oil objective (Nikon). Two spectra per pixel were acquired with an integration time of two seconds in steps of 0.5 µm in X and Y. Cosmic rays were removed automatically. The spectral fingerprint region of 600–1800 cm^−1^ was extracted from raw spectra, the 1^st^ derivative (size = 5, degree = 1) was calculated and unit vector normalization was performed. Principal component analysis (PCA) with three components was computed and displayed as a spectral map. Component spectra were shifted on the intensity scale for better visualization. The spectrum of Me_2_NNMe_2_ powder was acquired using the 532 nm laser at 100 mW, 2400 gr/mm grating, CFI Apo Lambda S ×40 NA 1.15 Water objective (Nikon) with 4 × 5 s integration and processed as described above. The processed spectrum of Me_2_NNMe_2_ was fitted to the spectral map of the cells by using the CLS function. All calculation and visualization steps were performed in LabSpec 6 (Horiba, Jobin Yvon, Bensheim, Germany).

### PDI reduction activity measurement

PDI reduction activity was measured using PROTEOSTAT PDI assay kit (#ENZ-51024, Enzo Life Sciences, Lausen, Switzerland). Experiments were performed according to the manufacturer’s instructions. Briefly, drugs alone or preincubated with CuCl_2_ (1:1) were added to a prepared insulin PDI solution. Then, DTT was added to start PDI reduction activity. After 30 min the reaction was stopped by the Stop reagent and the insulin precipitate was fluorescently labeled with Proteostat PDI detection reagent for 15 min. Fluorescence intensity was measured at 500 nm excitation and 603 nm emission using the spectrophotometer Tecan infinite 200Pro (Tecan Group, Männedorf, Switzerland).

### Glutathione measurement

Cells were plated (4 × 10^3^ cells/well) in 96-well plates and allowed to recover for 24 h. Then, cells were treated in sextuplicates with Triapine or Me_2_NNMe_2_ for another 24 h. Cells were lysed and levels of total and oxidized glutathione were measured in triplicates with GSH/GSSG-Glo^TM^ Assay (#V6611, Promega, Madison, USA) according to the manufacturer’s instructions. Fold increase in relative luminescence units (RLU) was calculated compared to untreated control after subtraction of cell-free blank.

### Detection of intracellular superoxide

Dihydroethidium (DHE, #D7008, Sigma-Aldrich, MO, USA) was used to detect the production of intracellular superoxide. Briefly, 5 × 10^5^ HL-60 cells per sample in 500 μl of PBS (78.1 mM Na_2_PO_4_ × 2 H_2_O, 14.7 mM KH_2_PO_4_, 26.8 mM KCl, 1.37 M NaCl) were incubated with indicated concentrations of Triapine and Me_2_NNMe_2_ for 45 min. Subsequently, DHE (10 µM) was added 15 min after the compounds. After incubation, the mean fluorescence intensity was measured by flow cytometry using a FACSCalibur instrument (Becton Dickinson, Palo Alto, CA, USA). Antimycin A (AMA, 10 µM) was used as positive control.

## Electronic supplementary material

Supplementary Table

Supplementary Figure 1

Supplementary Figure 2

Supplementary Figure 3

Supplementary Figure 4

Supplementary Figure 5

Supplementary Figure 6

Supplementary Figure 7

Supplementary Figure 8

Supplementary Figure 9

Supplementary Figure 10
