# Supplementary material for: The thiosemicarbazone Me2NNMe2 induces paraptosis by disrupting the ER thiol redox homeostasis based on protein disulfide isomerase inhibition
Source: Cell Death Dis. 2018 Oct 15;9(11):1052. doi: 10.1038/s41419-018-1102-z (PMC6189190; doi:10.1038/s41419-018-1102-z)
Supplement: Supplementary file 1 — Supplementary Table [file 41419_2018_1102_MOESM1_ESM.docx]

| **inhibitors** | **concentration of inhibitor** | **IC_50_-value (µM)** | |
| --- | --- | --- | --- |
|  |  | **Triapine** | **Me_2_NNMe_2_** |
| - | - | 0.586 ± 0.132 | 0.021 ± 0.015 |
| U0126 | 5 µM | 0.715 ± 0.061 | 0.367 ± 0.096 |
|  | 10 µM | 0.837 ± 0.097 | 0.412 ± 0.021 |
| PD98059 | 5 µM | 0.767 ± 0.107 | 0.162 ± 0.035 |
|  | 10 µM | 0.948 ± 0.340 | > 0.25 |
| trametinib | 100 nM | 0.667 ± 0.184 | > 0.25 |
| selumetinib | 50 nM | 0.688 ± 0.067 | > 0.25 |
| NAC | 1 mM | 0.622 ± 0.136 | > 0.5 |
|  | 2 mM | 0.732 ± 0.184 | > 0.5 |

**Suppl. Table**: IC_50_ values (72 h) of Triapine and Me_2_NNMe_2_ in SW480 cells with or without co-treatment of MEK inhibitors or the antioxidant NAC.

Hager et al. Suppl. Table
