# Supplementary figures and images for: The thiosemicarbazone Me2NNMe2 induces paraptosis by disrupting the ER thiol redox homeostasis based on protein disulfide isomerase inhibition
Source: Cell Death Dis. 2018 Oct 15;9(11):1052. doi: 10.1038/s41419-018-1102-z (PMC6189190; doi:10.1038/s41419-018-1102-z)

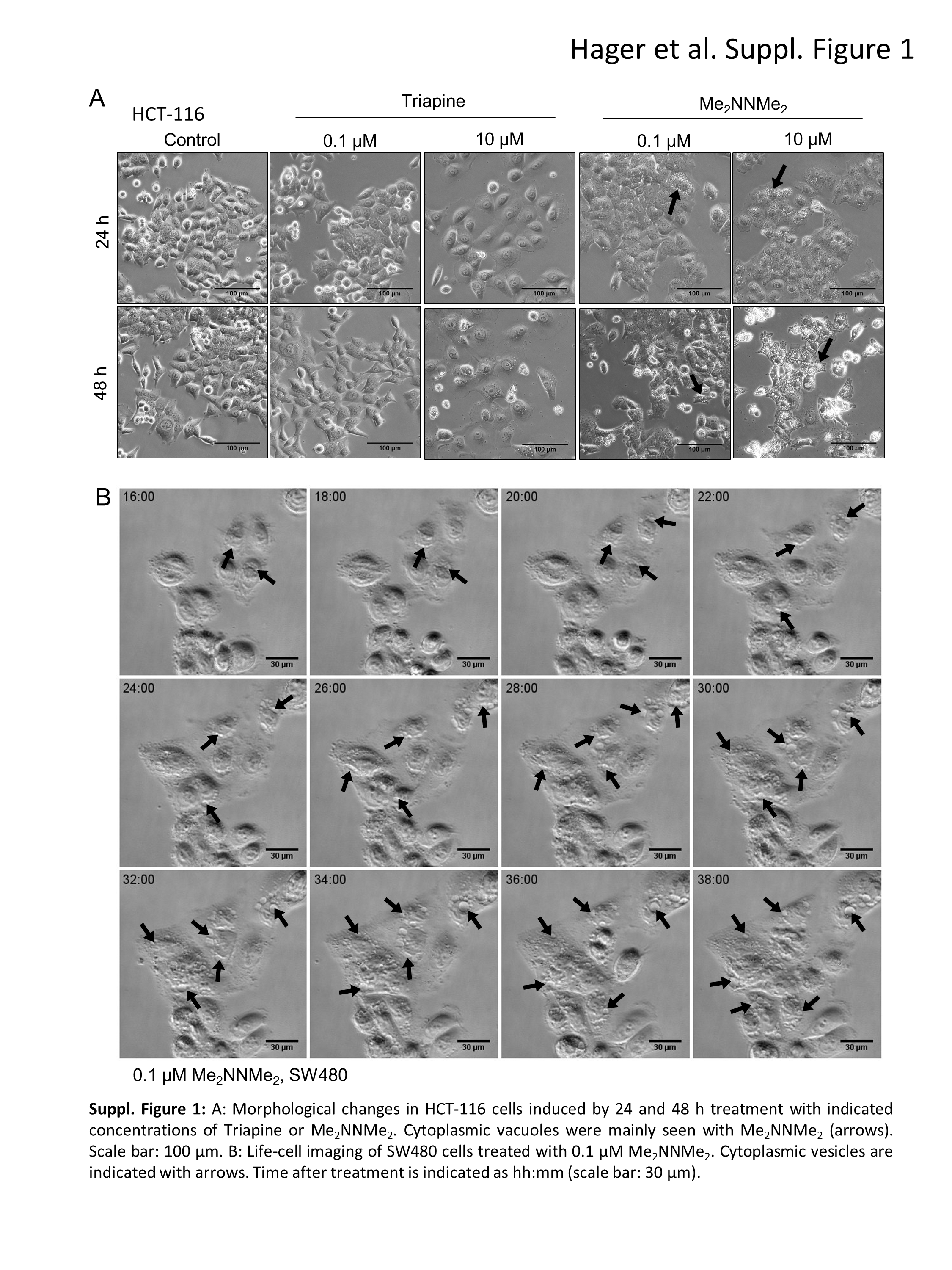

Supplement: Supplementary file 2 — Supplementary Figure 1 [file 41419_2018_1102_MOESM2_ESM.tif]

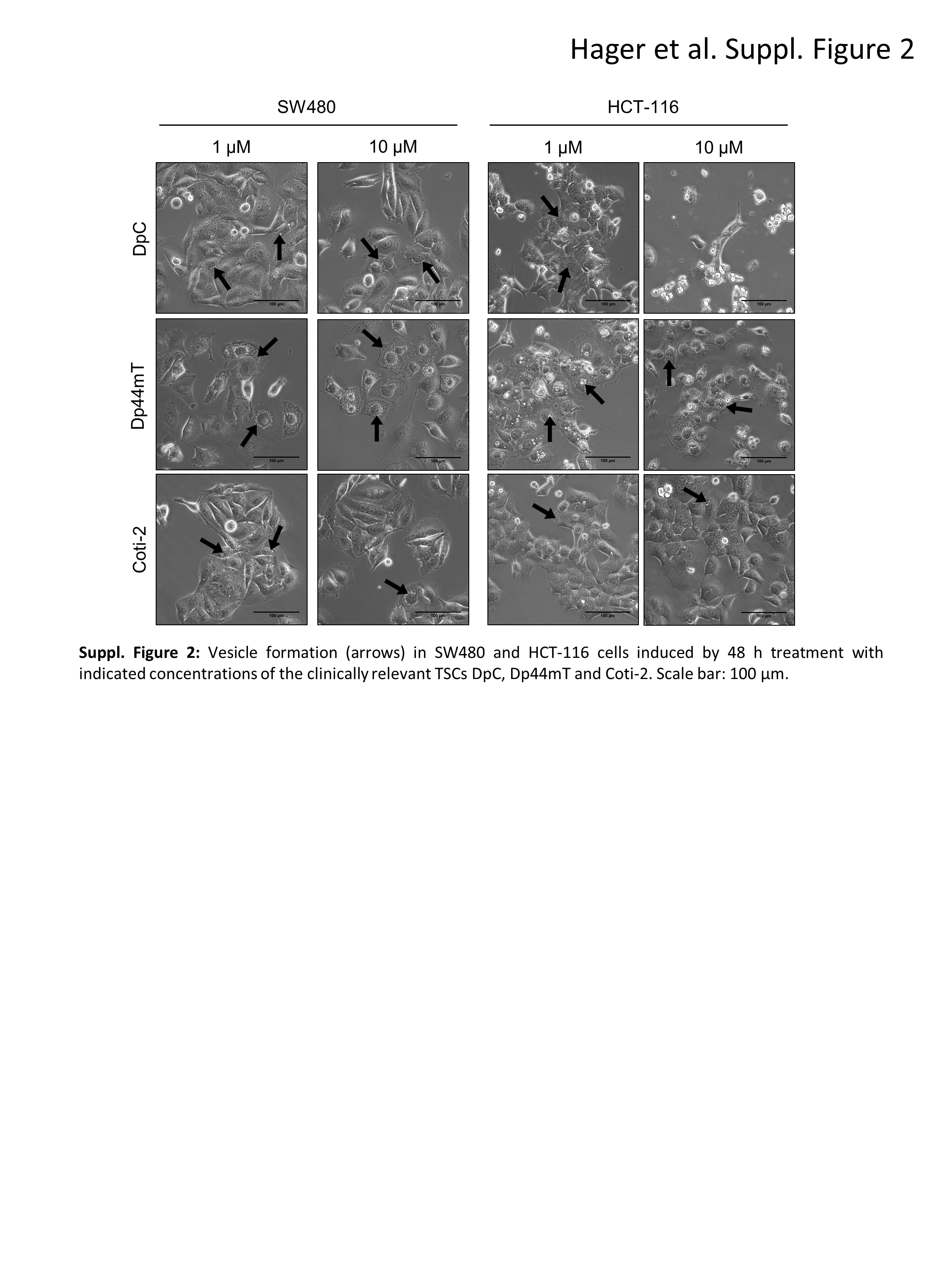

Supplement: Supplementary file 3 — Supplementary Figure 2 [file 41419_2018_1102_MOESM3_ESM.tif]

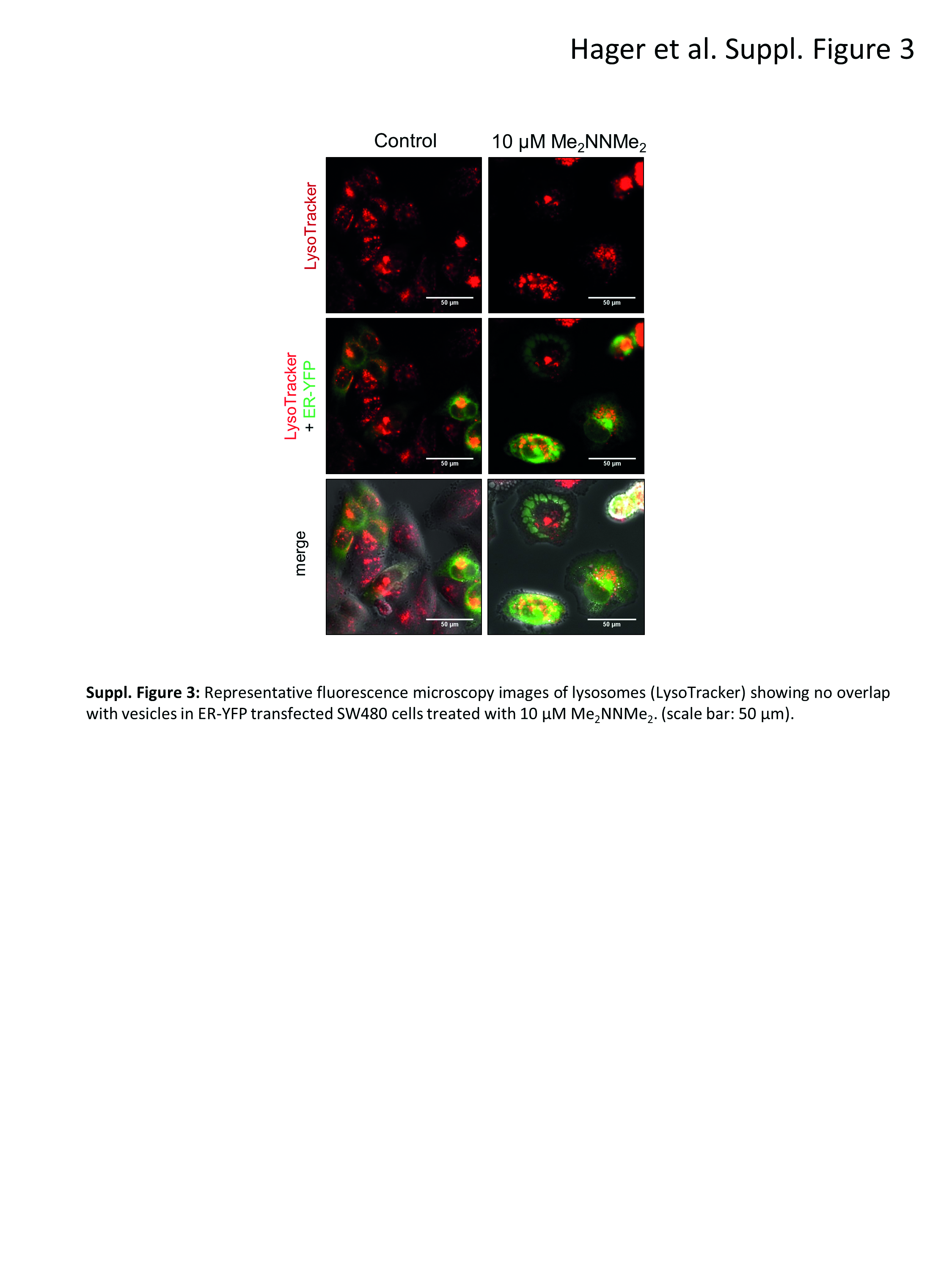

Supplement: Supplementary file 4 — Supplementary Figure 3 [file 41419_2018_1102_MOESM4_ESM.tif]

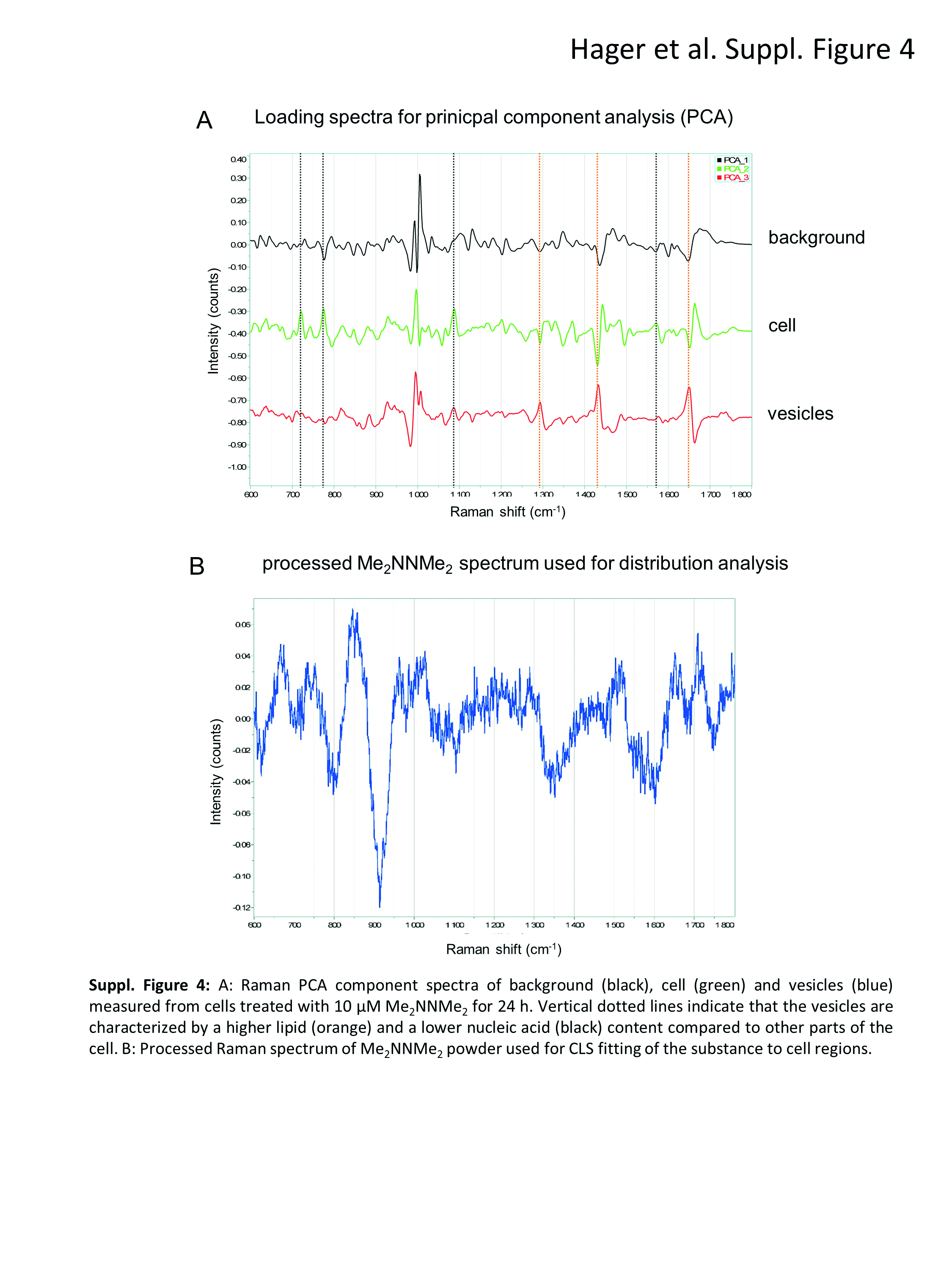

Supplement: Supplementary file 5 — Supplementary Figure 4 [file 41419_2018_1102_MOESM5_ESM.tif]

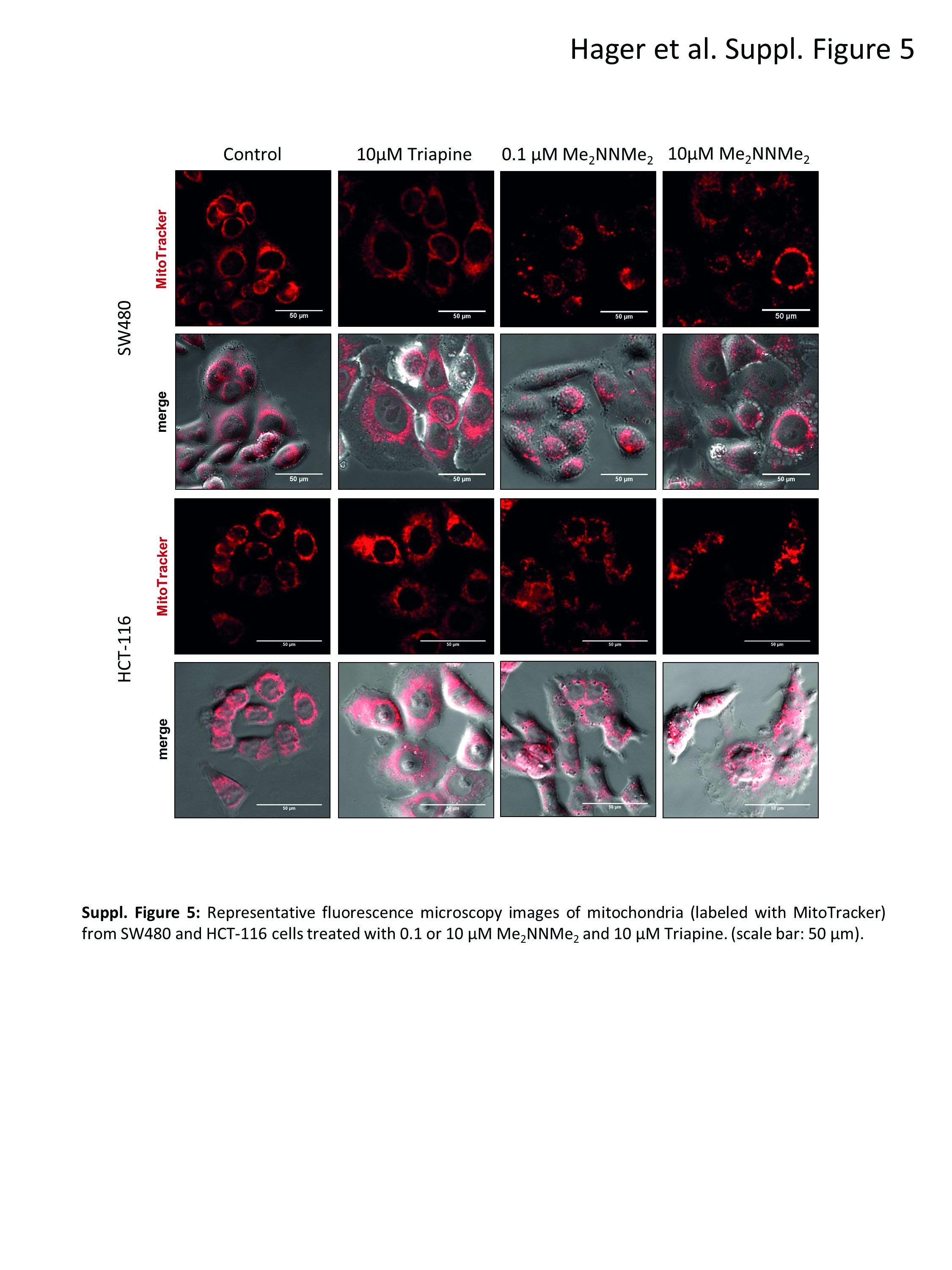

Supplement: Supplementary file 6 — Supplementary Figure 5 [file 41419_2018_1102_MOESM6_ESM.tif]

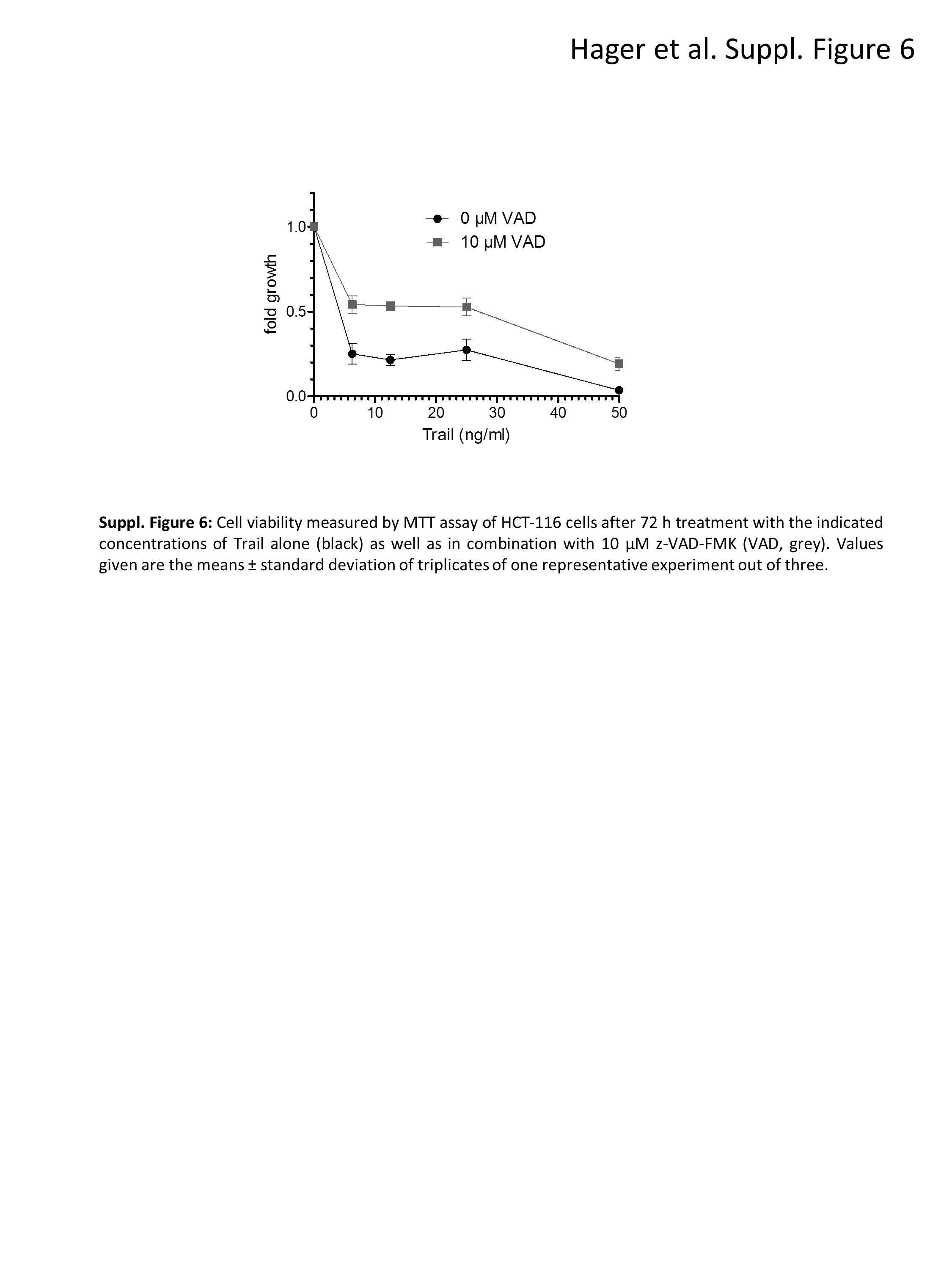

Supplement: Supplementary file 7 — Supplementary Figure 6 [file 41419_2018_1102_MOESM7_ESM.tif]

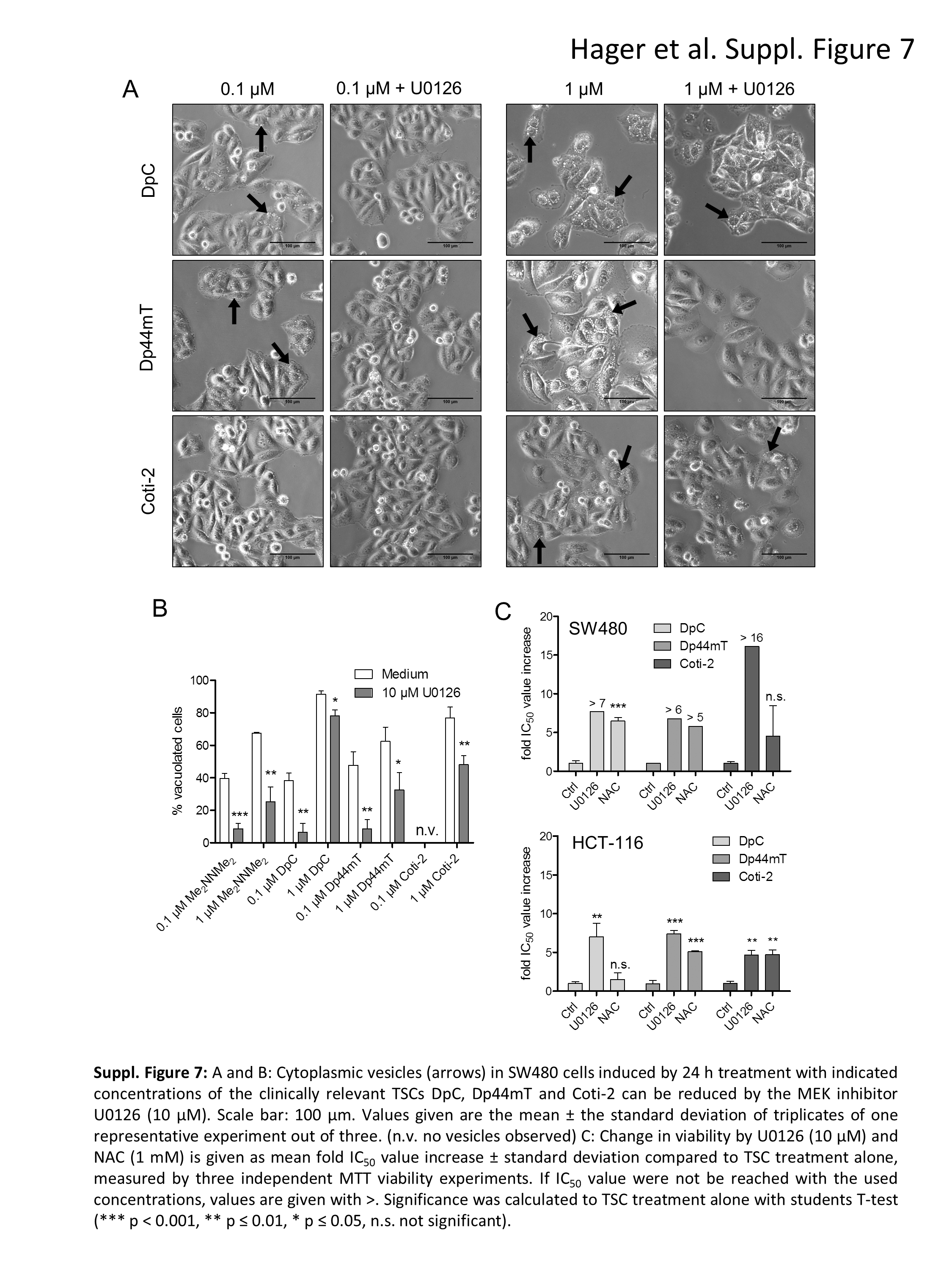

Supplement: Supplementary file 8 — Supplementary Figure 7 [file 41419_2018_1102_MOESM8_ESM.tif]

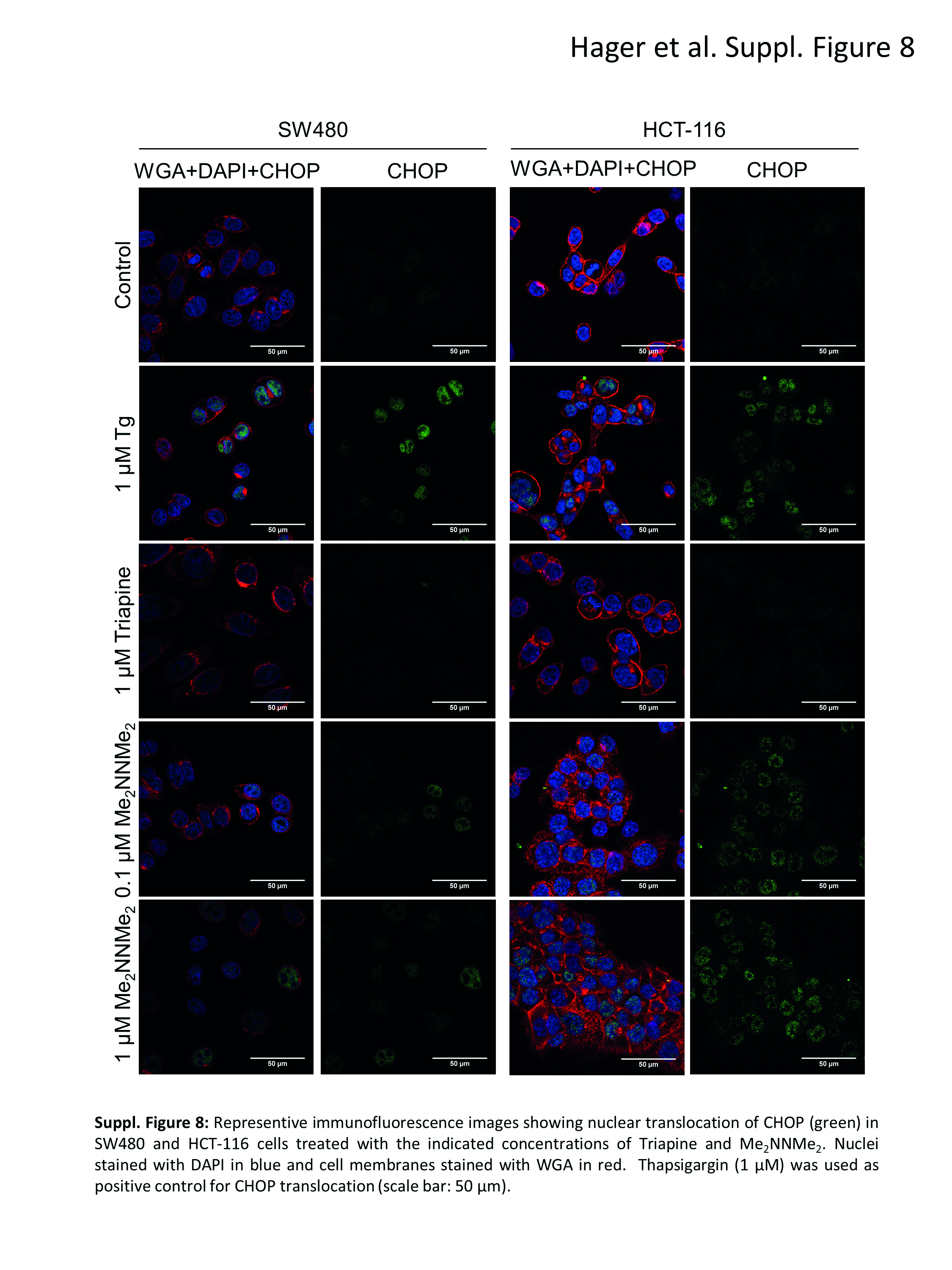

Supplement: Supplementary file 9 — Supplementary Figure 8 [file 41419_2018_1102_MOESM9_ESM.tif]

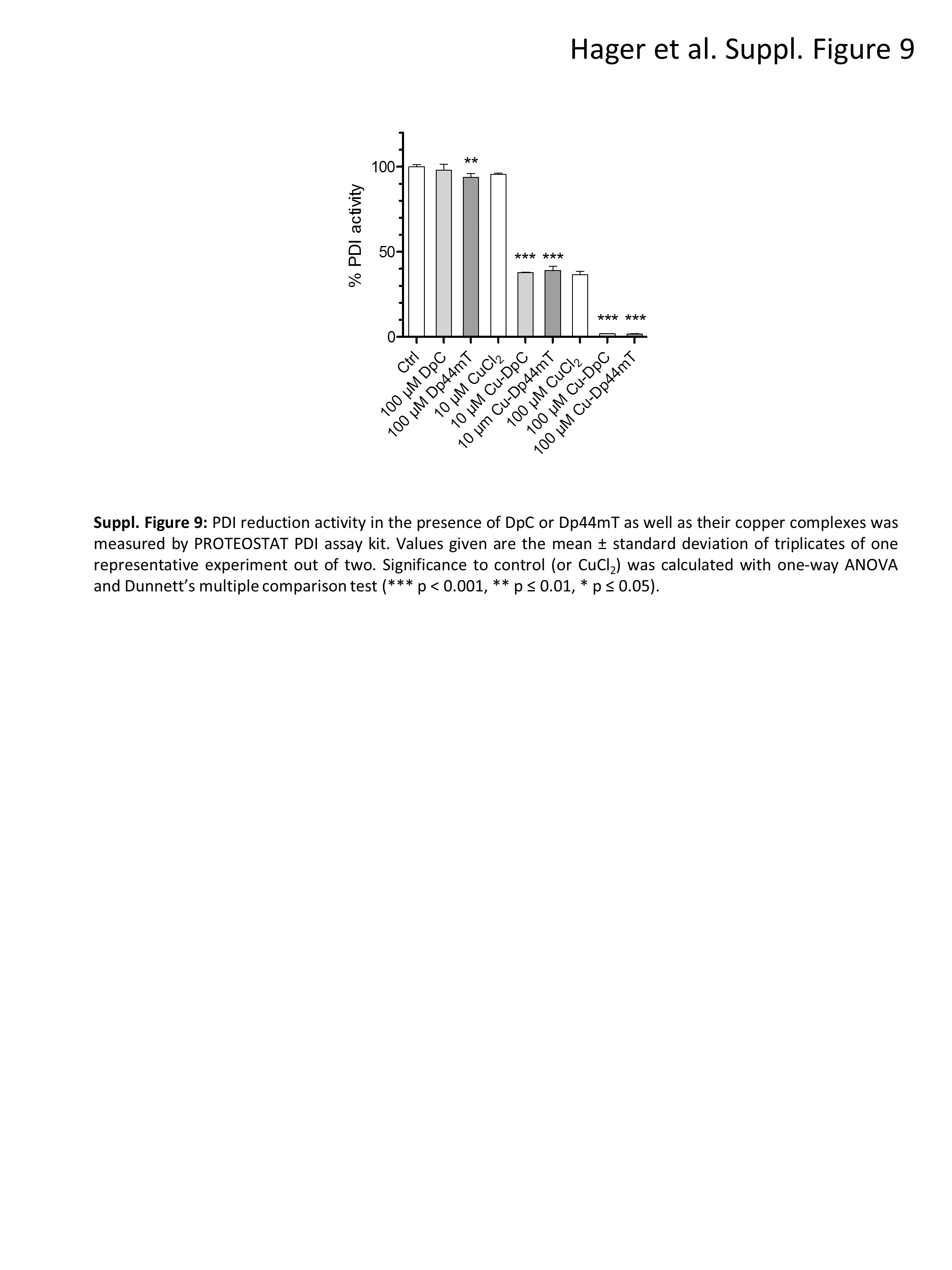

Supplement: Supplementary file 10 — Supplementary Figure 9 [file 41419_2018_1102_MOESM10_ESM.tif]

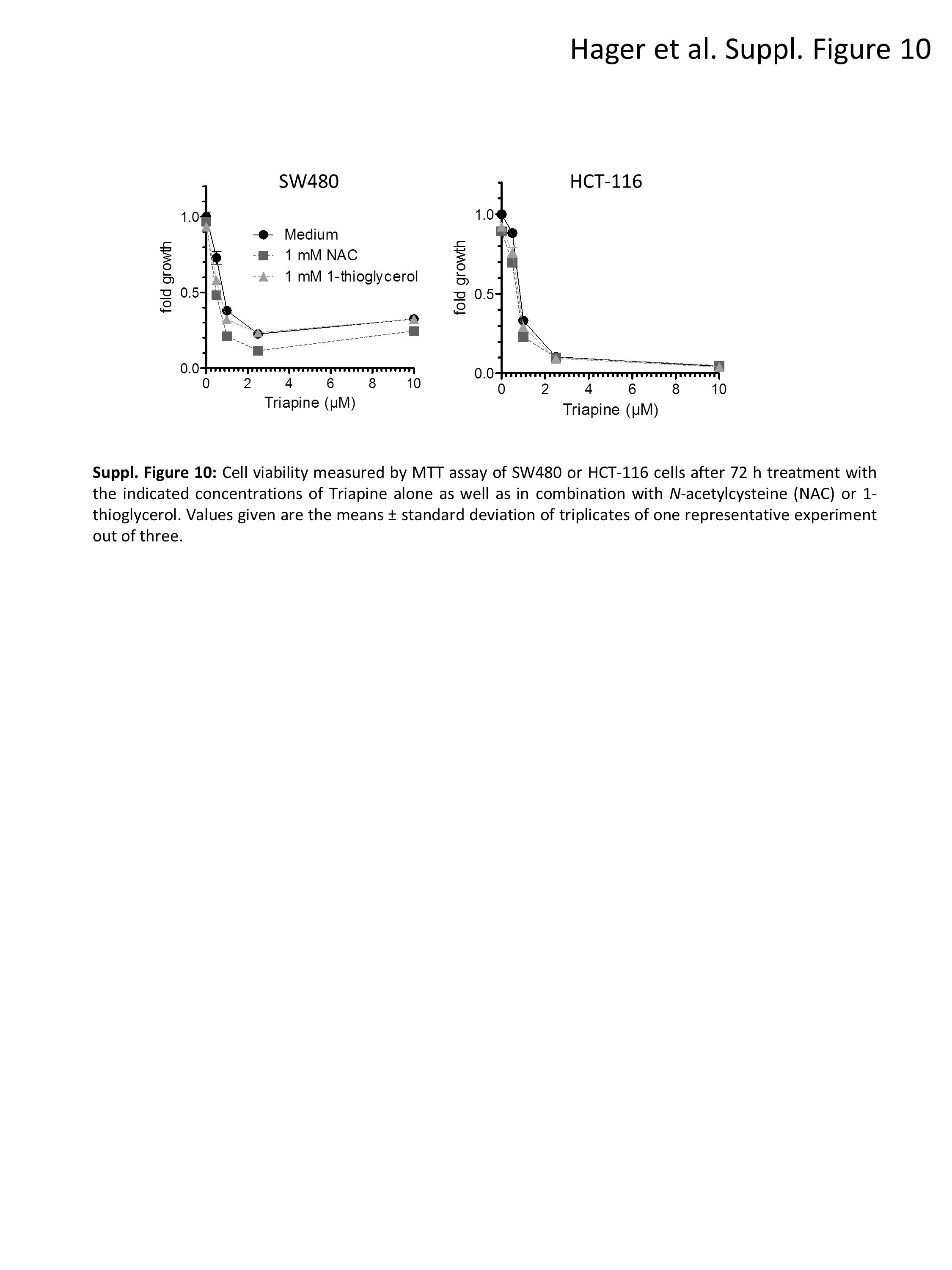

Supplement: Supplementary file 11 — Supplementary Figure 10 [file 41419_2018_1102_MOESM11_ESM.tif]
